# Digital Parenting Interventions for Fathers of Infants From Conception to the Age of 12 Months: Systematic Review of Mixed Methods Studies

**DOI:** 10.2196/43219

**Published:** 2023-07-26

**Authors:** Elisabeth Bailin Xie, James Wonkyu Jung, Jasleen Kaur, Karen M Benzies, Lianne Tomfohr-Madsen, Elizabeth Keys

**Affiliations:** 1 Department of Psychology University of Calgary Calgary, AB Canada; 2 Faculty of Nursing University of Calgary Calgary, AB Canada; 3 Department of Educational and Counselling Psychology, and Special Education The University of British Columbia Vancouver, BC Canada; 4 School of Nursing The University of British Columbia Okanagan Campus Kelowna, BC Canada

**Keywords:** eHealth, digital intervention, father, perinatal, infant, parenting, mobile phone, review

## Abstract

**Background:**

Digital interventions help address barriers to traditional health care services. Fathers play an important parenting role in their families, and their involvement is beneficial for family well-being. Although digital interventions are a promising avenue to facilitate father involvement during the perinatal period, most are oriented toward maternal needs and do not address the unique needs of fathers.

**Objective:**

This systematic review describes the digital interventions that exist or are currently being developed for fathers of infants from conception to 12 months postpartum.

**Methods:**

A systematic search of the MEDLINE, PsycINFO, Cochrane Central Register of Controlled Trials, Embase (using Ovid), and CINAHL (using EBSCO) databases was conducted to identify articles from database inception to June 2022, of which 39 met the inclusion criteria. Articles were included if they were peer-reviewed and described a digital intervention that targeted fathers of fetuses or infants aged ≤12 months. Systematic reviews, meta-analyses, and opinion pieces were excluded. Data from these studies were extracted and themed using a narrative synthesis approach. Quality appraisal of the articles was conducted using the Mixed Methods Appraisal Tool.

**Results:**

A total of 2816 articles were retrieved, of which 39 (1.38%) met the inclusion criteria for eligibility after removing duplicates and screening. Eligible articles included 29 different interventions across 13 countries. Most articles (22/29, 76%) described interventions that were exclusively digital. There were a variety of digital modalities, but interventions were most commonly designed to be delivered via a website or web-based portal (14/29, 48%). Just over half (21/39, 54%) of the articles described interventions designed to be delivered from pregnancy through the postpartum period. Only 26% (10/39) of the studies targeted fathers exclusively. A wide range of outcomes were included, with 54% (21/39) of the studies including a primary outcome related to intervention feasibility. Qualitative and mixed methods studies reported generally positive experiences with digital interventions and qualitative themes of the importance of providing support to partners, improving parenting confidence, and normalization of stress were identified. Of the 18 studies primarily examining efficacy outcomes, 13 (72%) reported a statistically significant intervention effect. The studies exhibited a moderate quality level overall.

**Conclusions:**

New and expecting fathers use digital technologies, which could be used to help address father-specific barriers to traditional health care services. However, in contrast to the current state of digital interventions for mothers, father-focused interventions lack evaluation and evidence. Among the existing studies on digital interventions for fathers, there seem to be mixed findings regarding their feasibility, acceptability, and efficacy. There is a need for more development and standardized evaluation of interventions that target father-identified priorities. This review was limited by not assessing equity-oriented outcomes (eg, race and socioeconomic status), which should also be considered in future intervention development.

## Introduction

### Background

Fathers play an important role within families during the transition to parenthood. Father involvement during the perinatal period (ie, from conception to 1 year following birth) and fathers’ access to knowledge about the transition to parenthood are important for perinatal mental health, improved adjustment to parenthood, and the provision of support from fathers to families [1]. New and expecting fathers are at risk of psychological distress in the perinatal period (eg, depression [2]), and these difficulties are associated with maternal postpartum depression [3,4], less and poorer quality of paternal involvement with their children’s development [5,6], and unhealthy lifestyle behaviors (eg, substance use [7]). Increased father involvement is associated with lower levels of depressive symptoms for fathers themselves [8]; better prenatal health behaviors, including decreased smoking and attaining prenatal care [9]; and early breastfeeding practices for mothers [10], as well as better neurodevelopment [11] and sleep duration and quality for infants [12,13]. Although all parenting partners and coparents play an important parenting role in the family, the World Health Organization has identified the importance of exploring effective strategies to increase the involvement of fathers in the process of pregnancy and childbirth to support mothers and their children, and there has been increasing recognition of the benefits of coparenting [14,15].

Research that aimed to investigate the needs and experiences of parents during the transition to parenthood found that fathers tend to report a lack of or inaccessible parenting information and supports specifically for fathers, as well as feelings of frustration about this lack of information in the antenatal period [16,17]. Resources targeted primarily toward mothers without being adapted for fathers can be ineffective as fathers’ needs can differ from those of mothers during the transition to parenthood [18,19]. Fathers have identified inflexible working practices, gaps in service, sleep deprivation, a lack of infant care skills, and feeling excluded by health professionals as specific barriers to receiving health information during the transition to parenthood [1,20]. Fathers have expressed the need and desire for access to relevant, accurate, and up-to-date information on infant care and challenges associated with new parenthood as well as the availability of support services [1,21]. Specifically, first-time fathers have described the need for more information on the demands of fatherhood shortly after birth, as well as how to recognize when to reach out for mental health supports [1]. In a video-modeled play intervention, fathers supported that 4 months postpartum was the right time to start the program, with some expressing interest in a higher frequency of home visits (ie, monthly) and having the intervention extended to a 1-year duration [21]. Moreover, the pandemic has been particularly burdensome for individuals in the transition to parenthood as it has unexpectedly altered the standard approaches to pregnancy and childbirth care, with many countries restricting access for fathers (ie, primary caregivers who are individuals who identify as male) and parenting partners (ie, any individual who supports the birthing parent) to attend medical appointments before and after the birth of their children [22]. Visitor restriction policies in hospitals were also found to be disproportionately harmful to racially diverse communities [23]. Thus, the pandemic has weakened the support offered to fathers, who have historically been less involved.

eHealth, which is the use of technology in the provision of health services [24,25], has become increasingly popular [26]. Extending eHealth to intervention delivery (referred to as *digital interventions* hereafter) helps address some of the common barriers associated with engaging with traditional health care services, such as accessibility, costs, availability, and time [1,27,28]. A key advantage of digital health interventions is their potential for scalability. During the COVID-19 pandemic, factors such as social and public health protections paired with additional childcare responsibilities for families and economic uncertainty contributed to increasingly challenging access to in-person services worldwide [29,30]. Parents often seek both information and support on the internet [31,32], and meta-analyses have shown that digital programs designed for parents are effective in improving parenting skills and child outcomes [33-35]. As such, complete or partial digital delivery of interventions has become increasingly beneficial during this time [36,37] and holds great potential for supporting fathers.

Although digital interventions exist for parents, they are often oriented toward mothers (ie, birthing parents or primary caregivers who are individuals who identify as female) rather than fathers and parenting partners or coparents [38,39], who have historically been underrepresented and excluded from parenting research [15]. For example, a recent systematic review of studies examining the efficacy of mobile interventions from conception to 1 year postpartum included fathers. In total, 3 of 12 studies including fathers were identified. Although the results suggested that both mothers and fathers benefited from the mobile interventions, the lack of data prevented the ability to draw conclusions regarding the relative mobile app effectiveness, and the authors suggested that future studies including fathers are needed [40]. In contrast to the growing popularity and promising findings of digital programs for mothers in the perinatal period [41-45], the authors of this review are not aware of any other reviews that have examined digital interventions (including any digital modalities) for fathers of young children.

Technology is widely used by new and expecting fathers as a source of parenting information, with fathers showing a strong interest in using internet-delivered strategies for mental health and parenting supports during the transition to fatherhood [46,47]. Although traditional health services, monitoring, and psychoeducation provided by health care clinicians are often key aspects of prenatal care [48], many fathers turn to digital technology to support them during their transition to fatherhood. For instance, fathers report enjoying and benefiting from listening to the stories of other fathers who have gone through similar experiences [49]. Web-based father support groups are being increasingly used by fathers, and as such, these are an important resource [47,50]. Although these types of informal support groups are valuable, evidence-based interventions that have been evaluated in controlled clinical trials with fathers specifically are crucial in ensuring effective support for fathers and, in turn, families in the transition to parenthood.

### Objectives

Father-targeted digital interventions are a promising avenue for promoting the health of families. The perinatal period is critical and time sensitive, wherein more paternal involvement and less maternal parenting stress can positively influence infant development [51]. Digital interventions offer a unique opportunity to re-examine how interventions can be more inclusive of fathers throughout the perinatal period to promote and maximize the benefits of father involvement. Therefore, this systematic review aimed to describe the digital interventions that exist or are currently being developed for fathers of infants—from conception to 12 months postpartum—to provide the foundation for future development, testing, and implementation of digital interventions for fathers. For the purpose of this review, *digital intervention* is defined as an intervention in which the digital component is crucial to program delivery (ie, the intervention could not have been delivered in an alternate format or an in-person portion was not substantial enough to be the only component of the intervention). In recognition of the unique roles and experiences of fathers [1,16,17,20], this review is focused on the term *fathers* rather than *parenting partners*.

## Methods

### Eligibility Criteria

This systematic review was conducted and reported in accordance with the PRISMA (Preferred Reporting Items for Systematic Reviews and Meta-Analyses) guidelines [52]. The articles had to describe a digital intervention that targeted fathers of fetuses and infants up to the age of 12 months. The inclusion and exclusion criteria for the papers are presented in Textbox 1.

**Inclusion and exclusion criteria for the papers.**

**Article type**

- Inclusion criteria: peer-reviewed articles or protocols
- Exclusion criteria: systematic reviews, meta-analyses, opinion pieces, conference proceedings, unpublished dissertations, books, and book pieces

**Language**

- Inclusion criteria: articles written in English, Spanish, Mandarin, Cantonese, French, or Punjabi
- Exclusion criteria: articles written in a language other than English, Spanish, Mandarin, Cantonese, French, or Punjabi

**Outcomes**

- Inclusion criteria: all outcomes included
- Exclusion criteria: none

**Age of the child**

- Inclusion criteria: fetuses and infants up to the age of 12 months and all infant populations (eg, infants born to term and preterm infants and those in hospital or the community)
- Exclusion criteria: children aged >12 months

**Intervention**

- Inclusion criteria: intervention described must have a digital component but did not have to be delivered exclusively using technology
- Exclusion criteria: no digital component or the digital component being too minor (ie, the intervention could have been conducted without the use of technology)

**Date of article publication**

- Inclusion criteria: all years
- Exclusion criteria: none

**Quality assessment of articles**

- Inclusion criteria: all quality ratings
- Exclusion criteria: none

**Pregnancy or delivery complications**

- Inclusion criteria: all included
- Exclusion criteria: none

### Search Strategy

The MEDLINE, PsycINFO, Cochrane Central Register of Controlled Trials, Embase (using Ovid), and CINAHL (using EBSCO) databases were used to identify studies from database inception to June 2022. The original search was conducted from April 2020 to May 2020 and identified 1614 studies, and the updated search from May 2022 to June 2022 identified the remaining 395 articles. Table 1 presents an example of the search strategy used, and Tables S1-S4 in Multimedia Appendix 1 present the search strategies for all other databases, which were drafted in consultation with an academic librarian. Following this search, a team of researchers—a PhD (EK), a PhD student (EBX), a Master of Sciences student (JWJ), and an undergraduate student (JK)—independently screened all titles and abstracts and full-text articles. In total, 2 researchers independently reviewed all abstracts and full texts, and additional authors (KMB and LTM; PhD) reviewed and resolved all conflicts that arose. Before extraction, protocol papers and trial registrations underwent a forward reference review by JWJ and JK to identify whether there was a subsequent published article following the protocol paper or trial registration. No backward reference review was conducted. Of the 39 studies included, 4 (10%) were added following the forward reference review (in lieu of the protocol or clinical trial registration that preceded it).

**Table 1:** Search strategy for the American Psychological Association PsycINFO database (date: May 15, 2020).

	Search terms	Records, n
1	exp telecommunications media/	18,448
2	exp computers/	42,174
3	exp mobile phones/	5205
4	eHealth.mp.	1011
5	e-Health.mp.	960
6	online.mp	87,483
7	on-line.mp.	7042
8	internet*.mp.	56,685
9	website*.mp.	15,159
10	computer*.mp.	145,381
11	mHealth.mp.	905
12	(smartphone* or “smartphone*”).mp.	4390
13	app.mp.	5944
14	apps.mp.	2000
15	“social media”.mp.	13,384
16	web-base*.mp.	13,436
17	mobile*.mp.	19,229
18	exp telemedicine/	8560
19	application*.mp.	188,003
20	exp mobile applications/	790
21	“cell* phone*”.mp.	2980
22	telehealth.mp.	1618
23	exp online social networks/	7639
24	exp social media/	14,030
25	exp computer games/	7436
26	“text messag*”.mp.	2712
27	SMS.mp.	1515
28	exp computer mediated communication/	15,619
29	1 or 2 or 3 or 4 or 5 or 6 or 7 or 8 or 9 or 10 or 11 or 12 or 13 or 14 or 15 or 16 or 17 or 18 or 19 or 20 or 21 or 22 or 23 or 24 or 25 or 26 or 27 or 28	454,785
30	infant*.mp.	118,011
31	baby.mp.	11,926
32	newborn*.mp.	11,396
33	babies.mp.	6340
34	“young child*”.mp.	40,827
35	infancy.mp.	19,196
36	neonate*.mp.	5794
37	NICU.mp.	1341
38	exp infant development/	21,698
39	30 or 31 or 32 or 33 or 34 or 35 or 36 or 37 or 38	176,107
40	exp fathers/	10,845
41	father*.mp.	50,046
42	paternal*.mp.	13,816
43	dad.mp.	698
44	dads.mp.	344
45	stepfather*.mp.	839
46	stepdad*.mp.	5
47	daddy.mp.	236
48	40 or 41 or 42 or 43 or 44 or 45 or 46 or 47	58,637
49	29 and 39 and 48	308

### Data Extraction and Synthesis

Extracted data for each intervention were tabulated according to the year of publication to assist in understanding any changes in intervention characteristics over time. Extracted data on study characteristics and the results of each study were tabulated according to the intervention’s primary topic of interest. Results were synthesized narratively. To assist with the synthesis of both qualitative and quantitative data, we used a parallel results convergent synthesis design, which consists of independent syntheses of quantitative and qualitative evidence with an interpretation of the results in the *Discussion* section [53,54]. Consistent with this approach, we analyzed and described the findings separately with some integration in the discussion because of limited qualitative research. For the qualitative studies with data on acceptability, feasibility, and usability, we used a textual narrative synthesis that involved dividing the studies into homogeneous groups and comparing similarities and differences across the studies [54]. Quantifiable data were tabulated, and frequency and percentages were calculated. Owing to the heterogeneity of the study designs, measures, and outcomes, we were unable to conduct inferential statistics. However, to understand the potential trends of the intervention characteristics on intervention effect, we further examined studies that used a randomized controlled trial (RCT) design by calculating the proportion of studies with particular intervention characteristics (ie, exclusively eHealth, digital or in-person human contact, and type of technology used) within studies that demonstrated significant intervention effects. This approach allowed the researchers to summarize a wide range of interventions and diverse study outcomes in a systematic way.

Pilot extraction was performed for reliability, and then extraction was completed independently by EK, EBX, JWJ, and JK. Data on study design, study aim, target population, intervention period, sample size, and study outcomes (eg, infant feeding, parenting knowledge, parenting self-efficacy, child outcomes, and parental mental health) were extracted and tabulated according to the primary intervention topic. Intervention descriptions were extracted and then characterized based on whether they were exclusively eHealth, meaning they were “stand-alone” and did not have a component that depended on an in-person interaction with a human provider, or a blended intervention, meaning that they had both digital and in-person components (eg, paper handouts, handbooks, newsletters, in-person parenting classes and parent support groups, and home visits by and discussions with health care professionals). Interventions were also categorized based on whether the intervention could be personalized to the participant’s needs and context or if it was standardized. Data on the types of digital modalities (eg, website, mobile app, SMS text messaging, social media, online video, email, or videoconference) that were included in each intervention were also tabulated. The favorability of the interventions was assessed and determined based on the reported acceptability, satisfaction and usability of the intervention or the rate of participant adherence and engagement. Interventions were categorized as favorable if there was an adherence or engagement rate of >60% or if >60% of the participants identified satisfaction, acceptability, or usability of the intervention. For studies with quantitative data on intervention effectiveness, statistically significant intervention effects were categorized based on *P* values (<.05).

Quality appraisals of each included article were conducted by the research team (EBX, JWJ, and JK) and verified by EK or KMB using the Mixed Methods Appraisal Tool (MMAT) [55]. The MMAT includes 2 screening questions (ie, whether there are clear research questions and whether the data allow for addressing the research question) and 5 quality criteria for each type of study design (ie, qualitative, quantitative RCT, quantitative nonrandomized, quantitative descriptive, and mixed methods). The questions in each domain are answered with “yes,” “no,” or “cannot tell.” The MMAT has been shown to be a reliable tool for reviews that incorporate diverse study designs [56] and provides overall methodological scores calculated as a percentage, from 0 (poor quality) to 100 (high quality). As in other reviews, scores were calculated as percentages based on the number of criteria met [57,58] to inform the quality of the studies and gauge the level of confidence in the study results. Mixed methods studies included both qualitative and the appropriate quantitative scores in their final MMAT score calculation.

## Results

### Overview

**Figure 1:**
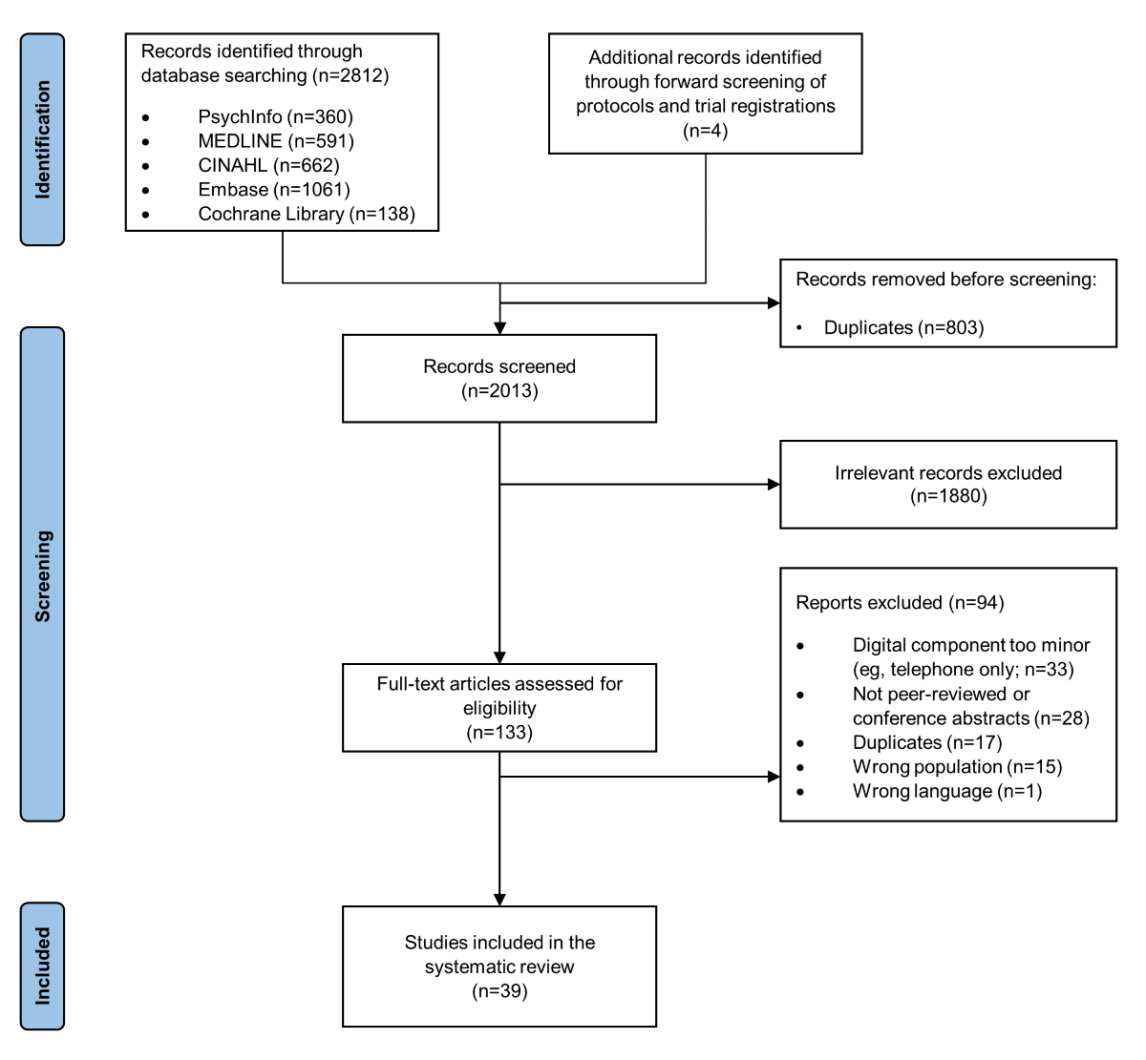
PRISMA (Preferred Reporting Items for Systematic Reviews and Meta-Analyses) flowchart of the included studies and exclusion reasons.

The search strategy retrieved 2816 articles, 803 (28.52%) of which were identified as duplicates, leaving the remaining 2013 (71.48%) titles and abstracts that were screened for eligibility. At the title and abstract screening stage, 93.39% (1880/2013) of the articles were excluded as irrelevant. Another 94 articles were excluded at full-text review, leaving the remaining 39 (29%) of the 133 studies that met the inclusion criteria and were included. Notably, 35% (33/94) of the articles were excluded from this review because the digital component was very minor, such as the use of DVDs, PowerPoint presentations, or videotape feedback that were reviewed in face-to-face sessions or phone calls (eg, [59-66]). Another 2% (2/94) of the articles were excluded because of the small percentage of fathers who were included in studies targeting couples (ie, 8.8% and 0.09% fathers; Figure 1) [67,68].

### Study Characteristics

Of the 39 peer-reviewed articles included, 1 (3%) described a protocol only; 1 (3%) described the development of the intervention as well as a study protocol; 9 (23%) described the development of the intervention only (ie, the intervention development was in progress); and 28 (72%) described the testing of the usability, satisfaction, or effect of an intervention. The studies included 29 unique interventions that were developed or tested in Canada (n=4, 14%), Brazil (n=1, 3%), the United States (n=7, 24%), Finland (n=1, 3%), China (n=2, 7%), Australia (n=6, 21%), Iran (n=2, 7%), Singapore (n=1, 3%), Turkey (n=1, 3%), South Korea (n=1, 3%), Denmark (n=1, 3%), Italy (n=1, 3%), and the Netherlands (n=1, 3%) between 2003 and 2022. The most common study designs were RCTs (14/39, 36%; 2/14, 14% of which were proposed studies) followed by quantitative nonrandomized studies (7/39, 18%). Table 2 provides more detailed descriptions of the study designs.

**Table 2:** Study characteristics (categorized by the topic of intervention development)^a,b^.

Study (author, year, country)		Study type	Study aim	Target population	Intervention period	Sample size
**Coparenting and partner support**						
	Pilkington et al [69], 2017, Australia	Pilot posttest–only single group—qualitative	Testing intervention usability	Mothers and fathers	Pregnancy and postpartum	5
	Firouzan et al [70], 2020, Iran	RCT^c^	Testing intervention efficacy	Fathers only	Pregnancy only	66 with 23 (35%) in the digital intervention group (in-person and SMS text message), 22 (33%) in the CD^d^ intervention group, and 21 (32%) in the control group
	Marcell et al [71], 2021, United States	Pilot RCT proposed	Intervention development (protocol paper)	Couples	Pregnancy and postpartum	120 with 60 (50%) in the intervention group and 60 (50%) in the control group^e^
**Parenting coping, satisfaction, and self-efficacy**						
	Hudson et al [72], 2003, United States	Pilot pre-post nonequivalent groups	Testing intervention efficacy	Fathers only	Postpartum only	34 with 14 (41%) in the intervention group and 20 (58%) in the control group
	Salonen et al [73], 2008, Finland	Cross-sectional observation of participants at 2 hospitals	Intervention development	Mothers and fathers	Pregnancy and postpartum	525 with 307 (58.5%) in the intervention group and 218 (41.5%) in the control group
	Salonen et al [74], 2011, Finland	Pre-post nonequivalent groups	Testing intervention efficacy	Mothers and fathers	Pregnancy and postpartum	436 with 53 (12.2%) in the nonuser intervention group, 149 (34.2%) in the user intervention group, and 234 (53.7%) in the control group
	Feinberg et al [75], 2020, United States	Pilot RCT	Testing intervention efficacy	Couples	Pregnancy and postpartum	36 with 15 (42%) in the intervention group and 21 (58%) in the control group
**Parenting skills and knowledge**						
	Fletcher et al [76], 2008, Australia	Pilot posttest–only single group	Intervention development	Fathers only	Pregnancy only	105
	Fletcher et al [77], 2016, Australia	Pilot posttest–only single group (mixed methods)	Intervention development and testing (quality and acceptability of SMS text messages)	Mothers and fathers	Postpartum only	67 with 46 (69%) in phase 2 (assessing acceptability) and 21 (31%) in phase 3 (message evaluation)
	Fletcher et al [78], 2017, Australia	Pilot posttest–only single group (mixed methods)	Testing intervention efficacy	Fathers only	Pregnancy and postpartum	46
	Fletcher et al [79], 2017, Australia	Pilot posttest–only single group	Testing intervention efficacy	Fathers only	Pregnancy and postpartum	520
	Mackert et al [80], 2017, United States	Pilot posttest–only single group (mixed methods)	Intervention development (investigate the value of the intervention)	Men only	Pregnancy only	23
	Lavin Venegas et al [81], 2019, Canada	Pilot RCT	Testing intervention efficacy	Mothers and fathers	Postpartum only	25 with 15 (60%) in the intervention group and 10 (40%) in the control group
	Fletcher et al [82], 2019, Australia	Single group, descriptive	Intervention development—asking EAG^f^ for feedback on content	Couples expected in intervention testing	Pregnancy and postpartum	14 (EAG members); 50 partners is the planned sample size for future testing^e^
	Fletcher et al [83], 2019, Australia	Posttest–only single group—qualitative	Testing mechanisms of impact of the intervention	Fathers only	Pregnancy and postpartum	40
	Fletcher et al [84], 2020, Australia	Pilot posttest–only single group—qualitative	Testing intervention feasibility	Couples	Pregnancy and postpartum	23
	Lanning et al [85], 2021, Australia	Posttest–only single group—qualitative	Development in progress	Couples	Pregnancy and postpartum	23
	Shorey et al [86], 2021, Singapore	Describing intervention development	Development in progress	Couples	Postpartum only	3 pairs of parents in the relevance cycle and another 10 people (including parents and research team members) for the evaluation cycle^g^
	Hägi-Pedersen et al [87], 2021, Denmark	Qualitative posttest–only single group	Testing intervention	Couples	Postpartum only	5
	Kavanagh et al [88], 2021, Australia	RCT	Testing intervention efficacy	Couples	Pregnancy and postpartum	124 with 62 (50.0 %) in each intervention group (treatment and active control)
**Infant feeding or breastfeeding**						
	White et al [39], 2016, Australia	Single group and single time point, including qualitative focus groups (mixed methods)	Intervention design, development, and pilot testing	Fathers only	Pregnancy and postpartum	22 with 18 (82%) in the focus group and 4 (18%) in the test group
	Abbass-Dick et al [89], 2017, Canada	Needs assessment and pre-post test	Intervention development and pilot efficacy testing	Couples	Pregnancy and postpartum	50 with 15 (30%) in phase 1 (needs assessment), 35 (70%) different individuals in phase 2 (efficacy); 24 (69%) fathers from phase 2 did phase 3 as well (satisfaction)
	White et al [90], 2018, Australia	Single-group qualitative analysis	Testing intervention use	Couples	Pregnancy and postpartum	586 with 208 (35.5%) in the contributor sample (those who posted on the forum at least once)
	White et al [91], 2019, Australia	Process evaluation	Describe process evaluation	Couples	Pregnancy and postpartum	400
	Abbass-Dick et al [92], 2020, Canada	RCT and mixed methods	Testing intervention efficacy	Couples	Pregnancy and postpartum	104 coparents with 50 (48.1%) in the intervention group and 54 (51.9%) in the control group. Coparents included male spouses (85.5%), same-sex spouses (1.8%), male partners (8.8%), maternal mother (2.7%), and friend (0.9%)
	Scott et al [93], 2021, Australia	RCT	Testing intervention efficacy	Couples	Pregnancy and postpartum	1092 with 299 (27.38%) in the digital intervention group, 263 (24.08%) in the face-to-face intervention group, 271 (24.82%) in the control group, and 259 (23.72%) in the combination group
**Parenting involvement**						
	Rhoads et al [94], 2015, United States	Posttest–only single group	Pilot feasibility testing	Mothers and fathers	Postpartum only	101
	Bonifacio et al [95], 2020, Brazil	Parallel cluster RCT	Testing intervention efficacy	Couples	Pregnancy and postpartum	186 with 62 (33.3%) in the digital intervention group, 73 (39.2%) in the nondigital intervention group, and 51 (27.4%) in the control group
**Injury prevention**						
	Yu et al [96], 2017, China	RCT	Testing intervention efficacy	Couples	Postpartum only	195 with 99 (50.7%) in the intervention group and 96 (49.2%) in the control group at 6 months and 97 (49.7%) in the intervention group and 93 (47.7%) in the control group at 12 months
**Mental health and well-being**						
	Da Costa et al [46], 2017, Canada	Needs assessment—descriptive	Intervention development	Fathers only	Pregnancy only	174
	Missler et al [97], 2020, Netherlands	RCT	Testing intervention efficacy	Couples	Pregnancy and postpartum	89 with 45 (51%) in the intervention group and 44 (49%) in the control group
	Zhang et al [98], 2021, China	RCT	Testing intervention efficacy	Couples	Postpartum only	84 couples with 42 (50%) in the intervention group and 42 (50%) in the control group^g^
**Parent-child relationship**						
	Benzies et al [99], 2013, Canada	RCT	Testing intervention efficacy	Fathers only	Postpartum only	111 with 46 (41.4%) in 2 visit intervention groups, 23 (20.7%) in 4 visit intervention groups, and 42 (37.8%) in the control group
	Manav et al [100], 2021, Turkey	RCT	Testing intervention efficacy	Couples	Postpartum only	32 fathers with 16 (50%) in the intervention group and 16 (50%) in the control group
	Doaltabadi and Amiri-Farahani [101], 2021, Iran	Pre-post nonequivalent groups (quasi-experimental study)	Testing intervention efficacy	Couples	Pregnancy only	114 with 38 (33.3%) in each digital intervention, face-to-face intervention, and control group
	Park and Bang [102], 2022, Korea	Quasi-experimental	Testing intervention efficacy	Fathers only	Postpartum only	32 with 15 (47%) in the intervention group and 17 (53%) in the control group
**Child health**						
	Whooten et al [103], 2021, United States	Describing the intervention and protocol for RCT	Intervention development and protocol for testing intervention efficacy	Couples	Pregnancy and postpartum	250 mother-father-infant triads, with 125 (50%) in the intervention group and 125 (50%) in the control group^e,g^
**NICU^h^ care**						
	Garfield et al [104], 2016, United States	Pilot RCT	Testing intervention efficacy	Couples	Postpartum only	41 with 20 (49%) in the intervention group and 21 (51%) in the control group
	Giuseppe et al [105], 2022, Italy	Prospective cohort pilot study	Testing intervention satisfaction and efficacy	Couples	Postpartum only	68 with 20 (29%) in the digital intervention group (Telematic-FCC^i^) and 24 (35%) in each of the other face-to-face comparison groups (FCC and no FCC)

^a^Articles are grouped by primary outcomes. However, many papers include outcomes that fit in various categories.

^b^There were mothers included in some studies but only reporting on father or partner sample size when provided.

^c^RCT: randomized controlled trial.

^d^CD: compact disc.

^e^The sample size reported refers to the planned sample size for the proposed future study.

^f^EAG: expert advisory group.

^g^Mother and father dyads combined in reporting.

^h^NICU: neonatal intensive care unit.

^i^FCC: family-centered care.

### Intervention Characteristics

In total, 31% (9/29) of the interventions clearly described a guiding theory (eg, self-efficacy theory or social cognitive theory) underlying intervention development or testing. Of the 29 different interventions, fathers appeared to have been involved in co-designing less than half (n=10, 34%). Co-design techniques included strategies such as father involvement in focus groups and interviews during app development (ie, the Milk Man app) [90,91,103], surveys of fathers on their information needs and factors associated with the decision to visit a father-focused website [46], and the incorporation of feedback from fathers during SMS text message development (ie, SMS4Dads) [77].

Most of the interventions (22/29, 76%) were exclusively digital. The remaining 24% (7/29) were blended interventions. The most commonly used digital component across the interventions was a website or web-based portal (14/29, 48%). Other digital technologies that were used included SMS text messages (7/29, 24%), mobile apps (7/29, 24%), digital videos (5/29, 17%), email (4/29, 14%), videoconferencing (4/29, 14%), and social media (1/29, 3%). Notably, the 14% (4/29) of interventions using videoconferencing modalities were recent, with publication dates of 2021 and 2022 [87,102,103,105]. Some interventions (11/29, 38%) included more than one type of eHealth component, such as the use of websites and email communication (3/29, 10%), SMS text messages, website resources and modules (2/29, 7%), or a mobile app and website links (2/29, 7%). A total of 3% (1/29) of the interventions used 4 digital modalities: SMS text messaging, web-based videos, email, and videoconferencing [103]. Multimedia Appendix 2 [39,46,69-114] provides more detailed intervention descriptions.

More than half (22/39, 56%) of the articles described an intervention that targeted couples together (eg, both mothers and fathers or coparents). Only 26% (10/39) of the studies targeted fathers exclusively, 3% (1/39) targeted men only, and the remaining 15% (6/39) included both mothers and fathers. Just over half (21/39, 54%) of the articles described an intervention that was designed to be delivered from pregnancy through the postpartum period, whereas 33% (13/39) targeted the postpartum period only, and 13% (5/39) targeted the prenatal period only.

### Quantitative Data Synthesis

When reviewing the study characteristics of interventions that had an effect (RCT design only; 13/29, 45%), 62% (8/13) detected intervention effects on a broad range of outcomes, including parenting knowledge, attitudes and confidence, parental mental health and perceived child sadness, father or partner prenatal engagement and presence during the birth, smoking cessation and exposure to secondhand smoke, and parent-child interaction quality. Of the 8 interventions that had significant results on the outcome of interest, 5 (62%) were exclusively eHealth (ie, were not part of a blended intervention with an in-person component) and 4 (50%) had a human intervention component (digital or in person). In terms of digital components, 60% (3/5) of the interventions that included a website had a significant effect, 75% (3/4) of the interventions that included SMS text messaging had a significant effect, 50% (2/4) of the interventions that included a mobile app had a significant effect, and only 33% (1/3) of those that used a web-based video had a significant effect. Of the 4 interventions that included more than one type of digital component, only 2 (50%) demonstrated a significant effect.

In terms of primary outcomes of the included studies, approximately half (21/39, 54%) had a primary outcome related to acceptability, usability, or satisfaction with the intervention. Of these 21 studies, 11 (52%) were examined for perceived favorability, with 8 (73%) being considered favorable by the review team and 3 (27%) being perceived to have unfavorable outcomes (see Table 3 for outcome summaries). The studies also evaluated intervention effects on outcomes such as coping, parental satisfaction, and parenting self-efficacy (12/39, 31%); coparenting (6/39, 15%); paternal mental well-being (6/39, 15%); parent-child relationship (5/39, 13%); infant feeding or breastfeeding (4/39, 10%); injury prevention (smoking cessation; 1/39, 3%); and infant physical health (weight gain; 1/39, 3%). Of the 18 studies primarily examining efficacy outcomes (ie, how effective the intervention was across various outcomes), 13 (72%) reported a statistically significant intervention effect. Statistically significant intervention effects were reported for father-child attachment and interaction [99,101,102]; knowledge about and attitudes toward participation in perinatal care [70]; smoking cessation and mothers’ secondhand smoking exposure [96]; parenting self-efficacy and satisfaction [72,74,104]; breastfeeding self-efficacy, knowledge, and infant feeding attitudes [89,92]; parental depression and child sadness [75]; and parental anxiety, depression, and quality of life [98].

Most of the included studies (31/39, 79%) used a convenience sample (ie, from clinics or health services, either self-selected or during a certain period, or a general convenience sample). In terms of study quality, MMAT scores ranged from 20% to 100%, with only 42% (15/36) of the articles meeting ≥80% of the MMAT criteria. In total, 25% (9/36) of the articles met all domain criteria, 17% (6/36) met 80% of the criteria, 28% (10/36) met 60% of the criteria, 19% (7/36) met 40% to 50% of the criteria, and 11% (4/36) met 20% of the criteria. A total of 8% (3/39) of the articles could not be appraised as they described protocols or processes [71,86,103]. Articles examining primary outcomes related to parent-child relationships, father mental health and well-being, injury prevention, and coparenting or partner support appeared to have slightly higher MMAT scores, with all scoring ≥60%. Articles examining primary outcomes related to parenting coping, satisfaction, self-efficacy, neonatal intensive care unit care, and parenting involvement appeared to have lower MMAT scores, with all scoring ≤60%. Articles examining primary outcomes related to infant feeding or breastfeeding and parenting skills and knowledge had a wider range of quality. Overall, a moderate level of confidence is expected from the results based on the quality of the studies included in the review, as indicated by the MMAT scores (Tables S1-S5 in Multimedia Appendix 3 [39,46,70,72-75,77,79,81-85,87-102,104,105,111]).

**Table 3:** Study results (categorized by the topic of intervention development)^a^.

Study (author, year)		Outcomes of interest	Finding notes
**Coparenting and partner support**			
	Pilkington et al [69], 2017	System quality, content quality, suggestions for website improvement, and potential barriers to visiting the website	>250 comments provided to inform changes to the intervention such as simplifying the language and structure of content and increasing the number of images. Barriers such as lack of time and smartphone incompatibility were identified.
	Firouzan et al [70], 2020	*Knowledge and attitudes about participation in perinatal care* ^b^	N/A^c^
	Marcell et al [71], 2021	Primary: infant knowledge, beliefs, self-efficacy, coparenting; secondary: partner relationship quality, infant care and engagement, time spent with infant, safe sleep, injury prevention care	N/A (protocol)
**Parenting coping, satisfaction, and self-efficacy**			
	Hudson et al [72], 2003	*Parenting self-efficacy, parenting satisfaction*, and satisfaction with the intervention	N/A
	Salonen et al [73], 2008	Parenting satisfaction and parenting self-efficacy	Found fathers experienced lowest self-efficacy related to the infant’s nutritional recommendations, day rhythm and sleep, normal development, and infant’s cues and behavior.
	Salonen et al [74], 2011	Parenting satisfaction (primary) and *self-efficacy (secondary)*	Parenting self-efficacy among all groups of fathers increased at 6 to 8 weeks postpartum.
	Feinberg et al [75], 2020	Parental efficacy and *depression* and relationship conflict, couples’ conflict, resolution style and *child sadness***,** child distress to limitations, and child soothability	N/A
**Parenting skills and knowledge**			
	Fletcher et al [76], 2008	Intervention usability, satisfaction, and uptake	≥95% agreed or strongly agreed that the package gave new information, that they intend to discuss the information with their partner, and that they are satisfied with the quality of the information; 78% agreed or strongly agreed that they did something differently because of this information^d^.
	Fletcher et al [77], 2016	Content clarity, usefulness, suitability, feasibility, and acceptability of the messages	90% indicated that the messages were easily understood and useful, and all participants easily identified which messages were targeted at fathers, mothers, or both. All transmitted messages were read by fathers and 74% felt that they were acceptable. Preferences toward messages that provided specific prompts and advice on ways to connect with and support their partner. Benefits also included prompting of discussions with their partners^d^.
	Fletcher et al [78], 2017	Intervention uptake, user engagement, acceptability, and psychological distress (mood tracker)	The most clicked link by fathers was “Becoming a dad: a big adjustment,” which received 22% (14/65) of all clicks, followed by “talking to your baby” (9/65, 14%). Out of those who responded to the Mood Tracker questions, half responded as “Cool” (47%) whereas 15% responded as “Shaky.” 87% of fathers remained engaged with the intervention, and interviews suggested that fathers were positive about their experience with the intervention^d^.
	Fletcher et al [79], 2017	Intervention uptake, user engagement and acceptability, psychological distress	63.1% clicked on at least one of the links provided. Links with the highest click rates were the Kidsafe NSW^e^ home safety checklist with 72.7%, Better Health’s “newborn screening” link at a 57.1% click rate, and the “alcohol pregnancy partner support” link from British Columbia’s Centre of Excellence for Women’s Health at a 50% click rate^d^.
	Mackert et al [80], 2017	Acceptability (general attitudes, actions, navigational issues, and technical trouble while testing out the intervention)	Participants agreed that it is important to know about pregnancy-related health information and expressed willingness to be involved in pregnancy but also reported feeling disconnected in the process. The theme of support for pregnant individuals emerged. Most participants (21/23, 91%) were engaged with the app, and the most clicked content was nutrition, followed by financial preparation^d^.
	Venegas et al [81], 2019	Study feasibility, acceptability of the video, and preliminary effectiveness (assessed by the use of any of the 3 pain management strategies—breastfeeding, skin-to-skin care, or sucrose) during NBS^f^	All parents in the intervention group viewed the full video and reported an intention to recommend the video to other parents. All but 2 parents reported that the video was the right length. Participants in the intervention group reported intentions to use or advocate for one of the pain management strategies. However, no significant difference between groups regarding the percentage of parents who used at least one pain management strategy^d^.
	Fletcher et al [82], 2019	Ratings on importance, clarity, evidence base, and the acceptability rating of intervention messages for mothers and fathers from EAG^g^ members and comments from the EAG group	For importance, clarity, evidence base, and acceptability, 81% of the messages met the requirement for fathers, according to the EAG. The EAG group provided feedback on the issues of grammar, the possibility of offending some individuals, and consideration of different relationship types^d^.
	Fletcher et al [83], 2019	Tested mechanisms of change (not outcomes of the intervention)—how it helped the men, specifically with respect to, becoming a father, their relationship with their infant, and their relationship with their partner	4 structural features identified—synced information, normalizing, prompts to interact and reflect, safety net, and 5 psychological processes identified—knowledge construction, confidence, ability to cope, role orientation, and feelings of connectedness.
	Fletcher et al [84], 2020	Uptake, user engagement, and acceptability	34.8% of fathers clicked on the website links in the messages; 25% clicked on the Mood Tracker links; 93% (15/16) were satisfied with the message frequency. Participants reported effects including increased knowledge about and interaction with their baby, normalization, and effective support for their partner^h^.
	Lanning et al [85], 2021	Qualitative assessment for identifying themes from the father’s experience partaking in this program.	Fathers found the messages to be helpful in the following areas during the interview: increased awareness that babies thrive on their connection with them, understanding the paternal role in the perinatal period, and that having an understanding via information received led to conversation and action.
	Shorey et al [86], 2021	Qualitative feedback assessing features, functionality, usability, and content accuracy	Pilot testing revealed technological and user issues, including web browser and app incompatibility, a lack of notifications, and limited search engine capability^i^.
	Hägi-Pedersen et al [87], 2021	Mothers’ and fathers’ experiences of the whole intervention trajectory	Interviews revealed an overarching theme of “oscillating between feeling confident in caring for the infant on your own and needing support from others.”
	Kavanagh et al [88], 2021	Program engagement and satisfaction, parenting efficacy (including putting baby to sleep), depression, quality of life, relationship satisfaction, social support, and self-efficacy for support provision^i^	Satisfaction with programs was high. However, only 20.9% of fathers accessed their assigned program more than once; 12.9% of fathers set a goal, and fathers accessed an average of 1 module. Partners experienced less relationship decline in the treatment group compared with the control group^h,i^.
**Infant feeding or breastfeeding**			
	White et al [39], 2016	Acceptability of the engagement strategies, appropriateness of the proposed approach and content, and mobile health app rating	Six areas for improvement in functionality and usability identified including text being too small, lack of clarity about how the points system worked, and the need for an important icon to be more prominent. The addition of a tutorial, options for users to post their own questions, and personalization of avatars were suggested.
	Abbass-Dick et al [89], 2017	*Breastfeeding self-efficacy, breastfeeding knowledge, infant feeding attitudes,* and perception of coparenting relationship and prototype usability	38% of fathers spent over 1 h reviewing the eHealth resource, and 67% of fathers strongly agreed that the resource was excellent overall^d^.
	White et al [90], 2018	Seek and offer support, social connection, and sharing experiences.	Themes for mobile app use included seeking and offering support, social connection, informational support provision, and sharing experiences.
	White et al [91], 2019	Participant app use and technology (software monitoring)^d^	Push notifications and interest in what other fathers had posted in the forum were the main motivators for mobile app use. Fathers used the app most while their partners were still pregnant and in the weeks immediately after the birth of their baby. At 6 weeks postpartum, approximately one-third of fathers still using the app said that the gamification elements were encouraging mobile app use.
	Abbass-Dick et al [92], 2020	Exclusive breastfeeding (primary) and *breastfeeding* duration, problems, self-efficacy, *knowledge,* partner support, coparenting, *infant feeding attitude*, intervention and breastfeeding resource use, and supplementation, satisfaction with an eHealth resource (secondary).	Both groups reported using generally available breastfeeding resources. Websites were used most often and rated as the most helpful. Breastfeeding partner support and coparenting scores were higher in the control group compared with the intervention group. For attitude and knowledge, there were no group differences at any follow-up time point, but scores increased more over time for the intervention group compared with the control group. Open-ended questions on satisfaction with the intervention were used to identify 5 themes^i^.
	Scott et al [93], 2021	Breastfeeding duration (primary) and age of formula or complementary food introduction, breastfeeding self-efficacy, and partner postpartum support (secondary)	N/A
**Parenting involvement**			
	Rhoads et al [94], 2015	Usability (number of logins to the web camera system, time spent viewing neonates—total number of minutes viewed, maximum time viewed in 1 login)	The mean number of logins for mothers was significantly greater than that for fathers (*P*=.03). There was no significant difference in mean total viewing time or maximum viewing time in 1 session.
	Bonifacio et al [95], 2020	Adherence to intervention and *partner attendance during prenatal care* and *presence at birth*	Partner adherence to the program was 53.4%^h^.
**Injury prevention**			
	Yu et al [96], 2017	*Smoking cessation* and *secondhand smoking exposure for mothers*	N/A
**Mental health and well-being**			
	Da Costa et al [46], 2017	Barriers to seeking help, men’s informational needs, user- and web-related factors associated with visiting a father-focused website	Fathers indicated wanting information on parenting and infant care, supporting, and improving the relationship with their partner, work-life balance, improving sleep, and managing stress. Important features of the website included it being personally relevant, credible, effective, and an easy navigation structure. Factors important for continued use were usefulness, readability, and being free of charge.
	Missler et al [97], 2020	Parenting stress (primary) and depression, anxiety, parental well-being (satisfaction with the parenting role, parenting self-efficacy, and sleep), parent-infant bonding, breastfeeding, room-sharing, infant crying, feeding, and sleeping (secondary)	N/A
	Zhang et al [98], 2021	*Anxiety, depression,* and *quality of life*	N/A
**Parent-child relationship**			
	Benzies et al [99], 2013	*Parent-child interaction***,** parental stress, and usability (number of booster dose videos viewed)	Fathers reviewed the videos in the web-based portal from 0 to 16 times; 71% of the fathers accessed at least 1 video over the 4 months of the study.
	Manav et al [100], 2021	Parent-infant attachment	Although attachment levels for fathers in both groups improved significantly over time, there were no significant differences between groups. There were significant effects on maternal attachment.
	Doaltabadi and Amiri-Farahani [101], 2021	*Father-infant attachment*	N/A
	Park and Bang [102], 2022	Knowledge of infant development, *father-infant interactions*, and father-infant attachment	N/A
**Child health**			
	Whooten et al [103], 2021	Prevalence of rapid infant weight gain (primary), WFL^j^, and prevalence of overweight. Maternal and paternal health behaviors, infant health behaviors, social and emotional well-being, family functioning, infant care, resource use, and COVID-19 pandemic impact	N/A
**NICU^k^ care**			
	Garfield et al [104], 2016	*Parenting self-efficacy* (primary) and *preparedness for discharge* and length of stay (secondary)	Significant within-group improvements in parenting self-efficacy but between-group differences were only significant when app use was accounted for in supplementary analyses^i^.
	Giuseppe et al [105], 2022	Satisfaction with adequate and timely information about the baby’s condition, with communication and collaboration with the health care team, and related to privacy and confidentiality, as well as *parental stress* (parental role alteration, infant appearance, and NICU environment)	Findings were mixed as statistics were provided for individual items of the scales rather than composite scores. Two out of 3 items related to satisfaction with communication and collaboration and 1 out of 3 items related to privacy showed that the FCC control group was better than the digital intervention. For the parental stressor scale, 11 out of 21 items showed that the FCC^l^ control was better than the T-FCC^m^ intervention, whereas 1 out of 21 items showed that the T-FCC intervention was better. Mothers reported more stress related to seeing tubes and IVs^n^ in their baby than fathers^i^.

^a^Articles are grouped by primary outcomes. However, many papers include outcomes that fit in various categories.

^b^Outcomes of interest in italics were found to be statistically significant. Finding notes provide additional detail when applicable and informative.

^c^N/A: not applicable.

^d^A favorable rating of intervention usefulness, satisfaction, and uptake based on results.

^e^NSW: New South Wales.

^f^NBS: newborn screening.

^g^EAG: expert advisory group.

^h^An unfavorable rating of intervention usefulness, satisfaction, and uptake based on results.

^i^Mother and father dyads combined in reporting.

^j^WFL: weight-for-length.

^k^NICU: neonatal intensive care unit.

^l^FCC: family-centered care.

^m^T-FCC: telematic–family-centered care.

^n^IV: intravenous.

### Qualitative Data Synthesis

The qualitative studies that were part of the review examined outcomes related to feasibility and usability as well as identifying findings related to user experience. Most articles (4/6, 67%) discussed interventions related to parenting skills and knowledge. In total, 50% (3/6) of the articles were on the SMS4Dads intervention, an intervention designed to deliver parenting skills and knowledge through SMS text messages and sometimes links to helpful websites [83-85]. The mixed methods articles similarly involved interventions targeting parenting skills and knowledge as well as infant feeding or breastfeeding. Most studies (4/5, 80%) examined outcomes related to the acceptability and feasibility of the intervention, with the exception of 20% (1/5) of the articles, which reported on intervention efficacy outcomes related to breastfeeding. All mixed methods studies included in this review involved interventions with a website component.

Overall, 40% (2/5) of the mixed methods articles described the aforementioned SMS4Dads intervention [77,78]. A common qualitative finding was the importance of providing support to partners during pregnancy and the postpartum period [77,80,84,90]. Another common qualitative finding involved the intervention helping fathers better understand their role and become more confident in their parenting [83,85,87]. A third common finding was the normalization of stress and fathers’ feelings of social connectedness in the intervention [83,84,90].

Of the studies that included a qualitative component (including the mixed methods studies), 55% (6/11) identified feedback from participants on their digital interventions. Participants generally reported having a positive experience with the digital interventions [77,78,92]. A total of 33% (2/6) of the articles similarly mentioned the importance of usability on mobile devices [69,92]. There was some discrepancy between the studies in terms of content. Participants in 17% (1/6) of the studies suggested simplifying the content included in the intervention [69], whereas participants in other studies reported wanting more specific solutions to specific problems [92] and suggested the inclusion of an additional tutorial [39]. Specific challenges with the digital interventions were also highlighted, such as the text being too small, wanting to personalize avatars, and content loading slowly, and a recommendation was made to make the intervention available during the prenatal period [39,92]. Finally, perceived barriers to father involvement included a lack of time and available resources [80,92]. Participants from 1 (17%) of these 6 studies suggested that making the intervention available during the prenatal period would provide fathers with more time compared with during the postpartum period [92].

In terms of MMAT quality ratings, the qualitative studies were of high quality, with 83% (5/6) of the articles meeting all criteria and 17% (1/6) that did not have an interpretation sufficiently substantiated by the data. For the mixed methods studies, 60% (3/5) did appear to have mixed methods designs but did not include an adequate rationale for using a mixed methods design to answer their research questions. No mixed methods studies included in this review met all the individual criteria for the quantitative and qualitative components involved in their methodology, which future mixed methods studies should consider in their study designs. These ratings suggest that we can have a high level of confidence in the findings of the qualitative studies. However, there is less confidence in the findings of the mixed methods studies.

## Discussion

### Principal Findings

To our knowledge, this is the first systematic review to describe studies on digital interventions (including any modalities) for fathers during the perinatal period. During the search, 39 articles were included that described 29 different interventions either under development or being tested across 13 different countries. There was a variety of digital components included in these interventions, classified into 7 distinct categories: web-based programs and websites, mobile apps, SMS text messages, digital videos, email, social media, and videoconferencing. On the basis of the MMAT appraisal, the articles included in this review demonstrated a range of quality levels (20%-100%), with a moderate quality level overall.

The interventions targeted a wide range of outcomes, including broader parenting outcomes (eg, self-efficacy, satisfaction, parent-child interaction, and infant knowledge) and father well-being as well as more specific outcomes related to breastfeeding and smoking cessation. However, most studies (21/39, 54%) focused on evaluating the feasibility, acceptability, and usability of the digital intervention or components of the intervention being developed. The emphasis on evaluating feasibility, acceptability, and usability highlights the growing interest in research on digital interventions for fathers of infants but also indicates the need for more rigorous research designs (ie, RCTs and high-quality mixed methods research designs) to determine if such interventions can result in improved health-related outcomes. With regard to feasibility and acceptability outcomes, of the 21 articles that examined them, just over half (12/21, 57%) provided basic descriptive results with no clear criteria for determining adequate feasibility or acceptability. To help address the lack of standardization in the evaluation of digital health interventions [115], future research on this topic should consider the use of well-established and validated measures such as the System Usability Scale [116]. The System Usability Scale is a 10-item questionnaire that can be used across various digital modalities, allowing for comparisons across interventions.

In studies that used an RCT design to evaluate the effect of the intervention on fathers’ knowledge, attitudes, behaviors, or health outcomes, the intervention topic did not appear to be related to whether the intervention was effective. Similarly, being an exclusively digital intervention or incorporating human interaction did not appear to be related to intervention effectiveness. There was also no clear trend in terms of which digital components were included in effective interventions, although it is notable that 75% (3/4) of the interventions that included SMS text messaging had a measurable impact on an outcome, whereas only 33% (1/3) of the interventions that included a web-based video had an effect. However, it is important to note that these results may not be generalizable because of the relatively small number of articles that could be compared.

The research team was surprised to discover that, despite being a common concern of new parents, there were no digital interventions that primarily targeted fathers’ sleep quality and quantity as study outcomes. This is consistent with a recent review highlighting that the role of fathers and other caretakers in infant sleep has been largely neglected [117]. This is problematic as sleep and mental health are challenges that have been expressed by new and expecting fathers and issues that have been identified as a patient-prioritized research gap from conception to the age of 24 months [118]. Fathers have identified wanting information related to managing sleep improvements [46]. Among the included studies, only 3% (1/29) of the interventions examined secondary father sleep outcomes [97], although there were no significant effects of this intervention on sleep outcomes. The only other studies that examined sleep focused on infant sleep outcomes, and they were also sparse. A total of 3% (1/29) of the interventions included a module on sleep and explored parenting self-efficacy items related to infant sleep; however, this was also nonsignificant [88]. Furthermore, an article that described intervention development examined levels of father self-efficacy related to infant sleep [73], and a protocol paper included safe sleep as a proposed secondary outcome [71]. Moreover, only 5% (2/39) of the studies primarily examined paternal mental health outcomes [97,98], one of which found significant intervention effects on mental health outcomes (parental anxiety and depression) [98]. In total, 5% to 15% of fathers in the perinatal period experience depression or anxiety, and perinatal illness contributes to adverse child and family outcomes, making paternal mental health an important target of intervention [119]. Additional development of digital interventions for fathers focused on mental health, which should include sleep as an important component of mental health, appears to be an underdeveloped area of research.

Few studies included in this review (11/39, 28%) measured or were planning to measure behavior change outcomes among fathers (ie, adjustments in behaviors, such as the implementation of parenting strategies, rather than a reported change in knowledge or attitudes). Although feasibility and fathers’ beliefs and knowledge about infant care are important, they may not necessarily lead to observable behavior change and parenting strategies, which are important for conferring secondary benefits for children and families [120,121]. For instance, Lavin Venegas et al [81] found that, although individuals in an intervention group designed to teach parents effective pain management strategies to use with their infants undergoing painful procedures reported intentions to use or advocate for the use of pain management strategies, there were no significant group differences in the actual use of pain management strategies. Therefore, future research should test interventions that target modifiable behavioral factors.

In contrast to the current state of the digital intervention literature for mothers in the perinatal period, digital intervention research for fathers lags behind, particularly with a lack of evidence regarding their effectiveness. For mothers, a systematic review suggested the effectiveness of digital tools in maternal health education, with a steady increase in studies in this area, particularly during the prenatal period, in the last decade [41]. Digital interventions have been shown to be effective in improving postpartum depression [42,43], treating insomnia during pregnancy [45], and preventing alcohol consumption [44]. There are mixed findings regarding their effectiveness for other mental health outcomes (eg, anxiety); psychosocial outcomes, including perceived stress, coping, and self-efficacy [42,122,123]; and physical health outcomes [124]. However, past reviews and meta-analyses suggest promising evidence, including cost-effectiveness [125], for the use of digital interventions for mothers during the perinatal period [123]. The focus of the literature on maternal-focused digital interventions seems to be on efficacy testing and implementation, whereas digital interventions for fathers appear to still be in their infancy, with more work being focused on intervention development and the feasibility and acceptability of these novel interventions. Given the effectiveness of digital interventions for mothers, pursuing this work with fathers is likely a worthwhile and promising avenue.

There may be continued challenges related to father recruitment and engagement in digital intervention research during the perinatal period. Only 26% (10/39) of the included studies (3/10, 30% of which tested the SMS4Dads intervention) [78,79,83] specifically recruited fathers, whereas the other studies recruited only couples or both. It is possible that traditional gender roles, such as fathers providing economically for their families and the stereotype that fathers are less involved in interactions with children compared with mothers, contribute to the relative absence of fathers in parenting research and the challenges with father recruitment and engagement [126].

Although there was limited qualitative research included in this review, the limited qualitative findings align with the quantitative results by suggesting that fathers are generally supportive of eHealth interventions and that they find such interventions to show promise in building parenting confidence and knowledge and promoting support toward partners. Qualitative findings also support the need to adapt digital interventions to accommodate father-specific needs and barriers. Suggestions from qualitative studies include mobile compatibility of web-based programs and making programs available during the prenatal period, when fathers may have more time compared with during the postpartum period.

Given the unique needs of fathers and their reported barriers to traditional health services [1,18-20], along with the potential for digital interventions to overcome these barriers, the development of digital interventions could be conducted specifically with fathers in mind. For instance, research can incorporate the use of patient advisory boards to ensure that the needs of patients are being met in a comprehensive way [127]. Past research has found couple-based interventions to be advantageous for parents as they can promote partner support of father involvement [15,128]. Although many of the current interventions are aligned with this as they target partners, future research could explore whether the effectiveness of interventions on fathers differs as a function of whether they are delivered solely to fathers or both partners simultaneously, specifically in a digital context.

### Limitations

This review is limited by the lack of data available in the existing literature on the assessment of how digital interventions may be differentially beneficial to various groups (eg, racial minority groups and those of low socioeconomic status). This is problematic as parents who are ethnically diverse and in lower socioeconomic groups tend to experience higher levels of parenting stress and conflict [129]. Furthermore, no studies described interventions for fathers in same-sex relationships or for gender-diverse individuals (with the exception of the study by Abbass-Dick et al [92]), which should be explored in future research and reviews. Additional research is needed to ensure that digital interventions are inclusive of all fathers and diverse family configurations.

### Future Directions

There appears to be a growing interest in the feasibility and acceptability of digital interventions for fathers of infants. Interventions for fathers have also been and are being developed to improve broad parenting abilities (eg, coparenting and parenting self-efficacy) and address specific topics (eg, smoking cessation and infant feeding). However, there is a need for more efficacy trial testing and also for interventions that target outcomes such as sleep and fathers’ mental health. Future research should also aim to improve the recruitment and engagement of fathers in perinatal studies as studies specifically examining fathers in parenting research were sparse and studies focusing primarily on mothers do not capture the full parenting picture. Furthermore, future studies should ensure that interventions are developed and tested in representative and generalizable samples. Interventions should aim to target father-identified priorities through direct partnerships with diverse patient populations [130].

### Conclusions

Leveraging digital technologies to develop and deliver interventions could help address barriers to traditional health care services that are specific to fathers. However, there seem to be mixed findings regarding the feasibility, acceptability, and efficacy of the existing digital interventions and the interventions under development. Future research on digital intervention development and testing is needed, and strategies to reach more fathers should be explored. Researchers may consider incorporating patient advisory boards to ensure that interventions address the specific needs of new and expecting fathers.

## Appendix

Multimedia Appendix 1 Search strategy tables.

Multimedia Appendix 2 Intervention descriptions organized by date of publication.

Multimedia Appendix 3 Mixed Methods Appraisal Tool quality assessment.

## Abbreviations

MMAT: Mixed Methods Appraisal Tool
PRISMA: Preferred Reporting Items for Systematic Reviews and Meta-Analyses
RCT: randomized controlled trial

## Data Availability

The data sets generated and analyzed during this study are available from the corresponding author upon reasonable request. This review was not registered, and a protocol was not published. Template data collection forms and the data that were extracted from the included studies and used in the analyses are available upon reasonable request from the corresponding author.

## References

[1] Baldwin S, Malone M, Sandall J, Bick D (2019). A qualitative exploratory study of UK first-time fathers' experiences, mental health and wellbeing needs during their transition to fatherhood. BMJ Open.

[2] Cameron EE, Sedov ID, Tomfohr-Madsen LM (2016). Prevalence of paternal depression in pregnancy and the postpartum: an updated meta-analysis. J Affect Disord.

[3] Paulson JF, Bazemore SD (2010). Prenatal and postpartum depression in fathers and its association with maternal depression: a meta-analysis. JAMA.

[4] Letourneau NL, Dennis C-L, Benzies K, Duffett-Leger L, Stewart M, Tryphonopoulos PD, Este D, Watson W (2012). Postpartum depression is a family affair: addressing the impact on mothers, fathers, and children. Issues Ment Health Nurs.

[5] Sethna V, Murray L, Netsi E, Psychogiou L, Ramchandani PG (2015). Paternal depression in the postnatal period and early father-infant interactions. Parent Sci Pract.

[6] Spector AZ (2006). Fatherhood and depression: a review of risks, effects, and clinical application. Issues Ment Health Nurs.

[7] Sipsma HL, Callands T, Desrosiers A, Magriples U, Jones K, Albritton T, Kershaw T (2016). Exploring trajectories and predictors of depressive symptoms among young couples during their transition to parenthood. Matern Child Health J.

[8] Bamishigbin ON, Wilson DK, Abshire DA, Mejia-Lancheros C, Schetter CD (2020). Father involvement in infant parenting in an ethnically diverse community sample: predicting paternal depressive symptoms. Front Psychiatry.

[9] Martin LT, McNamara MJ, Milot AS, Halle T, Hair EC (2007). The effects of father involvement during pregnancy on receipt of prenatal care and maternal smoking. Matern Child Health J.

[10] Bich TH, Hoa DT, Ha NT, Vui LT, Nghia DT, Målqvist M (2016). Father's involvement and its effect on early breastfeeding practices in Viet Nam. Matern Child Nutr.

[11] Jackson DB (2017). The interplay between early father involvement and neonatal medical risk in the prediction of infant neurodevelopment. Prev Sci.

[12] Keys EM, Benzies KM, Kirk VG, Duffett-Leger L (2022). Effect of Play2Sleep on mother-reported and father-reported infant sleep: a sequential explanatory mixed-methods study of a randomized controlled trial. J Clin Sleep Med.

[13] Bernier A, Tétreault É, Bélanger M-È, Carrier J (2016). Paternal involvement and child sleep. Int J Behav Dev.

[14] (2007). Male involvement in maternal health critical to saving women's lives, say UN leaders. United Nations Population Fund.

[15] Pilkington P, Rominov H, Brown HK, Dennis C-L (2019). Systematic review of the impact of coparenting interventions on paternal coparenting behaviour. J Adv Nurs.

[16] Deave T, Johnson D (2008). The transition to parenthood: what does it mean for fathers?. J Adv Nurs.

[17] Venning A, Herd MC, Smith DP, Lawn SJ, Mohammadi L, Glover F, Redpath P, Quartermain V (2021). “I felt like less than a shadow in the room”: systematic review of the experiences and needs of new fathers. Psychol Men Masc.

[18] Lechowicz ME, Jiang Y, Tully LA, Burn MT, Collins DA, Hawes DJ, Lenroot RK, Anderson V, Doyle FL, Piotrowska PJ, Frick PJ, Moul C, Kimonis ER, Dadds MR (2020). Enhancing father engagement in parenting programs: translating research into practice recommendations. Aust Psychol.

[19] Tully LA, Piotrowska PJ, Collins DA, Mairet KS, Black N, Kimonis ER, Hawes DJ, Moul C, Lenroot RK, Frick PJ, Anderson V, Dadds MR (2017). Optimising child outcomes from parenting interventions: fathers' experiences, preferences and barriers to participation. BMC Public Health.

[20] Shorey S, Ang L (2019). Experiences, needs, and perceptions of paternal involvement during the first year after their infants' birth: a meta-synthesis. PLoS One.

[21] Benzies KM, Magill-Evans J (2015). Through the eyes of a new dad: experiences of first-time fathers of late-preterm infants. Infant Ment Health J.

[22] Vasilevski V, Sweet L, Bradfield Z, Wilson AN, Hauck Y, Kuliukas L, Homer CS, Szabo RA, Wynter K (2022). Receiving maternity care during the COVID-19 pandemic: experiences of women's partners and support persons. Women Birth.

[23] Altman MR, Eagen-Torkko MK, Mohammed SA, Kantrowitz-Gordon I, Khosa RM, Gavin AR (2021). The impact of COVID-19 visitor policy restrictions on birthing communities of colour. J Adv Nurs.

[24] eHealth. World Health Organization.

[25] Oh H, Rizo C, Enkin M, Jadad A (2005). What is eHealth (3): a systematic review of published definitions. J Med Internet Res.

[26] Boogerd EA, Arts T, Engelen LJ, van de Belt TH (2015). "What is eHealth": time for an update?. JMIR Res Protoc.

[27] Mohr DC, Riper H, Schueller SM (2018). A solution-focused research approach to achieve an implementable revolution in digital mental health. JAMA Psychiatry.

[28] Carbonell Á, Navarro-Pérez J-J, Mestre M-V (2020). Challenges and barriers in mental healthcare systems and their impact on the family: a systematic integrative review. Health Soc Care Community.

[29] Brunier A, Drysdale C (2020). COVID-19 disrupting mental health services in most countries, WHO survey. World Health Organization.

[30] Núñez A, Sreeganga SD, Ramaprasad A (2021). Access to healthcare during COVID-19. Int J Environ Res Public Health.

[31] Daneback K, Plantin L (2008). Research on parenthood and the internet: themes and trends. Cyberpsychol J Psychosocial Res Cyberspace.

[32] Aston M, Price S, Hunter A, Sim M, Etowa J, Monaghan J, Paynter M (2021). Second opinions: negotiating agency in online mothering forums. Can J Nurs Res.

[33] Flannery J, Penner-Goeke L, Xie EB, Prince D, Simpson KM, Callaghan B, Tomfohr-Madsen L, Roos LE (2023). Digital parent training RCT meta-analysis and systematic review. PsyArXiv. Preprint posted online Jan 15, 2021.

[34] Spencer CM, Topham GL, King EL (2020). Do online parenting programs create change?: a meta-analysis. J Fam Psychol.

[35] Thongseiratch T, Leijten P, Melendez-Torres GJ (2020). Online parent programs for children's behavioral problems: a meta-analytic review. Eur Child Adolesc Psychiatry.

[36] Cheng Z, Mendolia S, Paloyo AR, Savage DA, Tani M (2021). Working parents, financial insecurity, and childcare: mental health in the time of COVID-19 in the UK. Rev Econ Househ.

[37] Fontanesi L, Marchetti D, Mazza C, Di Giandomenico S, Roma P, Verrocchio MC (2020). The effect of the COVID-19 lockdown on parents: a call to adopt urgent measures. Psychol Trauma.

[38] Plantin L, Daneback K (2009). Parenthood, information and support on the internet. A literature review of research on parents and professionals online. BMC Fam Pract.

[39] White BK, Martin A, White JA, Burns SK, Maycock BR, Giglia RC, Scott JA (2016). Theory-based design and development of a socially connected, gamified mobile app for men about breastfeeding (milk man). JMIR Mhealth Uhealth.

[40] Chua JY, Shorey S (2022). Effectiveness of mobile application-based perinatal interventions in improving parenting outcomes: a systematic review. Midwifery.

[41] Schnitman G, Wang T, Kundu S, Turkdogan S, Gotlieb R, How J, Gotlieb W (2022). The role of digital patient education in maternal health: a systematic review. Patient Educ Couns.

[42] Dol J, Richardson B, Murphy GT, Aston M, McMillan D, Campbell-Yeo M (2020). Impact of mobile health interventions during the perinatal period on maternal psychosocial outcomes: a systematic review. JBI Evid Synth.

[43] Zhou C, Hu H, Wang C, Zhu Z, Feng G, Xue J, Yang Z (2020). The effectiveness of mHealth interventions on postpartum depression: a systematic review and meta-analysis. J Telemed Telecare.

[44] Oh SS, Moon JY, Chon D, Mita C, Lawrence JA, Park E-C, Kawachi I (2022). Effectiveness of digital interventions for preventing alcohol consumption in pregnancy: systematic review and meta-analysis. J Med Internet Res.

[45] Silang KA, Sohal PR, Bright KS, Leason J, Roos L, Lebel C, Giesbrecht GF, Tomfohr-Madsen LM (2022). Ehealth interventions for treatment and prevention of depression, anxiety, and insomnia during pregnancy: systematic review and meta-analysis. JMIR Ment Health.

[46] Da Costa D, Zelkowitz P, Letourneau N, Howlett A, Dennis C, Russell B, Grover S, Lowensteyn I, Chan P, Khalifé S (2017). HealthyDads.ca: what do men want in a website designed to promote emotional wellness and healthy behaviors during the transition to parenthood?. J Med Internet Res.

[47] Cameron EE, Simpson KM, Pierce S, Penner KE, Beyak A, Gomez I, Bowes J-M, Reynolds K, Tomfohr-Madsen L, Roos L (2023). Paternal perinatal experiences during the COVID-19 pandemic: a framework analysis of the reddit forum Predaddit. Int J Environ Res Public Health.

[48] Benediktsson I, McDonald SW, Vekved M, McNeil DA, Dolan SM, Tough SC (2013). Comparing CenteringPregnancy® to standard prenatal care plus prenatal education. BMC Pregnancy Childbirth.

[49] Fletcher R, StGeorge J (2011). Heading into fatherhood--nervously: support for fathering from online dads. Qual Health Res.

[50] Eriksson H, Salzmann-Erikson M (2013). Supporting a caring fatherhood in cyberspace - an analysis of communication about caring within an online forum for fathers. Scand J Caring Sci.

[51] Kim M, Kang S-K, Yee B, Shim S-Y, Chung M (2016). Paternal involvement and early infant neurodevelopment: the mediation role of maternal parenting stress. BMC Pediatr.

[52] Page M, McKenzie J, Bossuyt P, Boutron I, Hoffmann T, Mulrow C, Shamseer L, Tetzlaff JM, Akl EA, Brennan SE, Chou R, Glanville J, Grimshaw JM, Hróbjartsson A, Lalu MM, Li T, Loder EW, Mayo-Wilson E, McDonald S, McGuinness LA, Stewart LA, Thomas J, Tricco AC, Welch VA, Whiting P, Moher D (2021). The PRISMA 2020 statement: an updated guideline for reporting systematic reviews. BMJ.

[53] Pluye P, Hong QN (2014). Combining the power of stories and the power of numbers: mixed methods research and mixed studies reviews. Annu Rev Public Health.

[54] Hong QN, Pluye P, Bujold M, Wassef M (2017). Convergent and sequential synthesis designs: implications for conducting and reporting systematic reviews of qualitative and quantitative evidence. Syst Rev.

[55] Hong QN, Pluye P, Fàbregues S, Bartlett G, Boardman F, Cargo M, Dagenais P, Gagnon M-P, Griffiths F, Nicolau B, O’Cathain A, Rousseau M-C, Vedel I (2018). Mixed Methods Appraisal Tool (MMAT), version 2018. Registration of Copyright (#1148552), Canadian Intellectual Property Office, Industry Canada.

[56] Pace R, Pluye P, Bartlett G, Macaulay AC, Salsberg J, Jagosh J, Seller R (2012). Testing the reliability and efficiency of the pilot Mixed Methods Appraisal Tool (MMAT) for systematic mixed studies review. Int J Nurs Stud.

[57] Baillot A, Chenail S, Barros Polita N, Simoneau M, Libourel M, Nazon E, Riesco E, Bond DS, Romain AJ (2021). Physical activity motives, barriers, and preferences in people with obesity: a systematic review. PLoS One.

[58] Mącznik AK, Ribeiro DC, Baxter GD (2015). Online technology use in physiotherapy teaching and learning: a systematic review of effectiveness and users' perceptions. BMC Med Educ.

[59] Raouna A, Malcolm R, Ibrahim R, MacBeth A (2021). Promoting sensitive parenting in 'at-risk' mothers and fathers: a UK outcome study of Mellow Babies, a group-based early intervention program for parents and their babies. PLoS One.

[60] Chen Y-L, Lee T-Y, Gau M-L, Lin K-C (2019). The effectiveness of an intervention program for fathers of hospitalized preterm infants on paternal support and attachment 1 month after discharge. J Perinat Neonatal Nurs.

[61] Kadivar M, Mozafarinia SM (2013). Supporting fathers in a NICU: effects of the hug your baby program on fathers' understanding of preterm infant behavior. J Perinat Educ.

[62] Ihekuna D, Rosenburg N, Menson WN, Gbadamosi SO, Olawepo JO, Chike-Okoli A, Cross C, Onoka C, Ezeanolue EE (2018). Male partner involvement on initiation and sustainment of exclusive breastfeeding among HIV-infected post-partum women: study protocol for a randomized controlled trial. Matern Child Nutr.

[63] Keys E, Benzies KM, Kirk V, Duffett-Leger L (2018). Using play to improve infant sleep: a mixed methods protocol to evaluate the effectiveness of the Play2Sleep intervention. Front Psychiatry.

[64] Nosrati A, Mirzakhani K, Golmakani N, Esmaeily H, Nekah SM (2019). Effect of attachment training on paternal-fetal attachment. J Midwifery Reprod Health.

[65] Giallo R, Seymour M, Fogarty A, Hosking C, Williams LA, Cooklin A, Grobler A, Ride J, Leach L, Oldenburg B, Wood C, Borschmann R, O'Brien J, Evans K, Treyvaud K, Garfield C, Brown S, Nicholson J (2022). Working out dads (WOD): a study protocol for a randomised controlled trial of a group-based peer support intervention for men experiencing mental health difficulties in early fatherhood. BMC Psychiatry.

[66] Phianching K, Chaimongkol N, Pongjaturawit Y (2020). Effects of the parental sensitivity intervention among mothers and fathers of preterm infants: a quasi-experimental study. Pac Rim Int J Nurs Res.

[67] Brown C, Davis KE, Habiba N, Massey-Stokes M, Warren C (2020). Parent preferences for text messages containing infant feeding advice. Mhealth.

[68] Helle C, Hillesund ER, Wills AK, Øverby NC (2019). Evaluation of an eHealth intervention aiming to promote healthy food habits from infancy -the Norwegian randomized controlled trial Early Food for Future Health. Int J Behav Nutr Phys Act.

[69] Pilkington PD, Rominov H, Milne LC, Giallo R, Whelan TA (2016). Partners to Parents: development of an online intervention for enhancing partner support and preventing perinatal depression and anxiety. Adv Ment Health.

[70] Firouzan V, Noroozi M, Mirghafourvand M, Farajzadegan Z (2020). Comparing the effect of group- based training along with text messaging and compact disc- based training on men's knowledge and attitude about participation in perinatal care: a cluster randomized control trial. BMC Pregnancy Childbirth.

[71] Marcell AV, Johnson SB, Nelson T, Labrique AB, Van Eck K, Skelton S, Aqil A, Gibson D (2021). Protocol for the feasibility, acceptability, and preliminary efficacy trial of text4father for improving underserved fathers' involvement in infant care. J Health Care Poor Underserved.

[72] Hudson DB, Campbell-Grossman C, Fleck MO, Elek SM, Shipman A (2003). Effects of the New Fathers Network on first-time fathers' parenting self-efficacy and parenting satisfaction during the transition to parenthood. Issues Compr Pediatr Nurs.

[73] Salonen AH, Kaunonen M, Astedt-Kurki P, Järvenpää A-L, Tarkka M-T (2008). Development of an internet-based intervention for parents of infants. J Adv Nurs.

[74] Salonen AH, Kaunonen M, Astedt-Kurki P, Järvenpää A-L, Isoaho H, Tarkka M (2011). Effectiveness of an internet-based intervention enhancing Finnish parents' parenting satisfaction and parenting self-efficacy during the postpartum period. Midwifery.

[75] Feinberg ME, Boring J, Le Y, Hostetler ML, Karre J, Irvin J, Jones DE (2019). Supporting military family resilience at the transition to parenthood: a randomized pilot trial of an online version of family foundations. Fam Relat.

[76] Fletcher R, Vimpani G, Russell G, Keatinge D (2008). The evaluation of tailored and web-based information for new fathers. Child Care Health Dev.

[77] Fletcher R, May C, Wroe J, Hall P, Cooke D, Rawlinson C, Redfern J, Kelly B (2016). Development of a set of mobile phone text messages designed for new fathers. J Reprod Infant Psychol.

[78] Fletcher R, May C, Lambkin F-K, Gemmill AW, Cann W, Nicholson JM, Rawlinson C, Milgrom J, Highet N, Foureur M, Bennett E, Skinner G (2017). SMS4dads: providing information and support to new fathers through mobile phones – a pilot study. Adv Mental Health.

[79] Fletcher R, Kay-Lambkin F, May C, Oldmeadow C, Attia J, Leigh L (2017). Supporting men through their transition to fatherhood with messages delivered to their smartphones: a feasibility study of SMS4dads. BMC Public Health.

[80] Mackert M, Guadagno M, Lazard A, Donovan E, Rochlen A, Garcia A, Damásio MJ (2017). Engaging men in prenatal health promotion: a pilot evaluation of targeted e-health content. Am J Mens Health.

[81] Lavin Venegas C, Taljaard M, Reszel J, Dunn S, Graham I, Harrold J, Larocque C, Nicholls B, Nicholls S, OʼFlaherty P, Squires J, Stevens B, Trépanier M-J, Harrison D (2019). A parent-targeted and mediated video intervention to improve uptake of pain treatment for infants during newborn screening: a pilot randomized controlled trial. J Perinat Neonatal Nurs.

[82] Fletcher R, Campbell L, Sved Williams A, Rawlinson C, Dye J, Baldwin A, May C, StGeorge J (2019). SMS4 perinatal parents: designing parenting support via text messages for mothers with severe mental illness (SMI) and their partners. Adv Mental Health.

[83] Fletcher R, Knight T, Macdonald JA, StGeorge J (2019). Process evaluation of text-based support for fathers during the transition to fatherhood (SMS4dads): mechanisms of impact. BMC Psychol.

[84] Fletcher R, StGeorge JM, Rawlinson C, Baldwin A, Lanning P, Hoehn E (2020). Supporting partners of mothers with severe mental illness through text - a feasibility study. Australas Psychiatry.

[85] Lanning P, Rawlinson C, Hoehn E, De Young A, StGeorge J, Fletcher R (2022). Primary mental health prevention in partners of mothers with a major mental illness: SMS4Dads. J Reprod Infant Psychol.

[86] Shorey S, Tan TC, Mathews J, Yu CY, Lim SH, Shi L, Ng ED, Chan YH, Law E, Chee C, Chong YS, thilagamangai (2021). Development of a supportive parenting app to improve parent and infant outcomes in the perinatal period: development study. J Med Internet Res.

[87] Hägi-Pedersen MB, Kronborg H, Norlyk A (2021). Knowledge of mothers and fathers' experiences of the early in-home care of premature infants supported by video consultations with a neonatal nurse. BMC Nurs.

[88] Kavanagh DJ, Connolly J, Fisher J, Halford WK, Hamilton K, Hides L, Milgrom J, Rowe H, Scuffham PA, White KM, Wittkowski A, Appleton S, Sanders D (2021). The baby steps web program for the well-being of new parents: randomized controlled trial. J Med Internet Res.

[89] Abbass-Dick J, Xie F, Koroluk J, Alcock Brillinger S, Huizinga J, Newport A, Goodman WM, Dennis C-L (2017). The Development and piloting of an eHealth breastfeeding resource targeting fathers and partners as co-parents. Midwifery.

[90] White BK, Giglia RC, Scott JA, Burns SK (2018). How new and expecting fathers engage with an app-based online forum: qualitative analysis. JMIR Mhealth Uhealth.

[91] White B, Giglia RC, White JA, Dhaliwal S, Burns SK, Scott JA (2019). Gamifying breastfeeding for fathers: process evaluation of the Milk Man mobile app. JMIR Pediatr Parent.

[92] Abbass-Dick J, Sun W, Newport A, Xie F, Godfrey D, Goodman WM (2020). The comparison of access to an eHealth resource to current practice on mother and co-parent teamwork and breastfeeding rates: a randomized controlled trial. Midwifery.

[93] Scott JA, Burns SK, Hauck YL, Giglia RC, Jorgensen AM, White BK, Martin A, Robinson S, Dhaliwal SS, Binns CW, Maycock BR (2021). Impact of a face-to-face versus smartphone app versus combined breastfeeding intervention targeting fathers: randomized controlled trial. JMIR Pediatr Parent.

[94] Rhoads S, Green A, Gauss CH, Mitchell A, Pate B (2015). Web camera use of mothers and fathers when viewing their hospitalized neonate. Adv Neonatal Care.

[95] Bonifácio LP, Franzon AC, Zaratini FS, Vicentine FB, Barbosa-Júnior F, Braga GC, Sanchez JA, Oliveira-Ciabati L, Andrade MS, Fernandes M, Fabio SV, Duarte G, Pileggi VN, Souza JP, Vieira EM (2020). PRENACEL partner - use of short message service (SMS) to encourage male involvement in prenatal care: a cluster randomized trial. Reprod Health.

[96] Yu S, Duan Z, Redmon PB, Eriksen MP, Koplan JP, Huang C (2017). mHealth intervention is effective in creating smoke-free homes for newborns: a randomized controlled trial study in China. Sci Rep.

[97] Missler M, van Straten A, Denissen J, Donker T, Beijers R (2020). Effectiveness of a psycho-educational intervention for expecting parents to prevent postpartum parenting stress, depression and anxiety: a randomized controlled trial. BMC Pregnancy Childbirth.

[98] Zhang Q-L, Lei Y-Q, Liu J-F, Cao H, Chen Q (2021). Using telemedicine to improve the quality of life of parents of infants with CHD surgery after discharge. Int J Qual Health Care.

[99] Benzies KM, Magill-Evans J, Kurilova J, Nettel-Aguirre A, Blahitka L, Lacaze-Masmonteil T (2013). Effects of video-modeling on the interaction skills of first-time fathers of late preterm infants. Infants Young Child.

[100] Manav AI, Gozuyesil E, Tar E (2021). The effects of the parenting education performed through Whatsapp on the level of maternal-paternal and infant attachment in Turkey. J Pediatr Nurs.

[101] Doaltabadi Z, Amiri-Farahani L (2021). The effect of in-person and virtual prenatal care education of the spouses of primiparous women on the father and mother's attachment to infant: a quasi-experimental and controlled study. Trials.

[102] Park S-E, Bang K-S (2022). Effects of a hybrid online and offline program for facilitating father-infant interactions in South Korea: a quasi-experimental study. Child Health Nurs Res.

[103] Whooten RC, Kwete GM, Farrar-Muir H, Cournoyer RN, Barth EA, Kotelchuck M, Taveras EM (2021). Engaging fathers in the first 1000 days to improve perinatal outcomes and prevent obesity: rationale and design of the First Heroes randomized trial. Contemp Clin Trials.

[104] Garfield CF, Lee YS, Kim HN, Rutsohn J, Kahn JY, Mustanski B, Mohr DC (2016). Supporting parents of premature infants transitioning from the NICU to home: a pilot randomized control trial of a smartphone application. Internet Interv.

[105] Giuseppe DB, Giuseppina N, Desiree S, Angela S, Maurizio G, Perrone S (2022). Improving care in neonatal intensive units during the COVID-19 pandemic: a survey on electronic health communication. J Intensive Care Med.

[106] Bandura A (1997). Self Efficacy: The Exercise of Control.

[107] Bronfenbrenner U (2005). Making Human Beings Human Bioecological Perspectives on Human Development.

[108] Dowrick PW (2012). Self model theory: learning from the future. Wiley Interdiscip Rev Cogn Sci.

[109] Bandura A (1995). Self-Efficacy in Changing Societies.

[110] Bandura A (1986). Social Foundations of Thought and Action a Social Cognitive Theory.

[111] Fletcher R, May C, Attia J, Garfield CF, Skinner G (2018). Text-based program addressing the mental health of soon-to-be and new fathers (SMS4dads): protocol for a randomized controlled trial. JMIR Res Protoc.

[112] Glanz K, Rimer BK, Lewis FM (1990). Health Behavior and Health Education Theory, Research, and Practice.

[113] Bandura A (1977). Self-efficacy: toward a unifying theory of behavioral change. Psychol Rev.

[114] Bowlby J (1983). Attachment: Second Edition.

[115] Inal Y, Wake JD, Guribye F, Nordgreen T (2020). Usability evaluations of mobile mental health technologies: systematic review. J Med Internet Res.

[116] Brooke J (1996). SUS: a 'quick and dirty' usability scale. Usability Evaluation In Industry.

[117] Reuter A, Silfverdal S-A, Lindblom K, Hjern A (2020). A systematic review of prevention and treatment of infant behavioural sleep problems. Acta Paediatr.

[118] Bright KS, Ginn C, Keys EM, Brockway ML, Tomfohr-Madsen L, Doane S, Benzies K (2018). Study protocol: determining research priorities of young Albertan families (The Family Research Agenda Initiative Setting Project-FRAISE)-participatory action research. Front Public Health.

[119] Darwin Z, Domoney J, Iles J, Bristow F, Siew J, Sethna V (2020). Assessing the mental health of fathers, other co-parents, and partners in the perinatal period: mixed methods evidence synthesis. Front Psychiatry.

[120] Tavassolie T, Dudding S, Madigan AL, Thorvardarson E, Winsler A (2016). Differences in perceived parenting style between mothers and fathers: implications for child outcomes and marital conflict. J Child Fam Stud.

[121] Neel ML, Stark AR, Maitre NL (2018). Parenting style impacts cognitive and behavioural outcomes of former preterm infants: a systematic review. Child Care Health Dev.

[122] MacKinnon AL, Silang K, Penner K, Zalewski M, Tomfohr-Madsen L, Roos LE (2022). Promoting mental health in parents of young children using eHealth interventions: a systematic review and meta-analysis. Clin Child Fam Psychol Rev.

[123] Sakamoto JL, Carandang RR, Kharel M, Shibanuma A, Yarotskaya E, Basargina M, Jimba M (2022). Effects of mHealth on the psychosocial health of pregnant women and mothers: a systematic review. BMJ Open.

[124] Hussain T, Smith P, Yee LM (2020). Mobile phone-based behavioral interventions in pregnancy to promote maternal and fetal health in high-income countries: systematic review. JMIR Mhealth Uhealth.

[125] Carrandi A, Hu Y, Karger S, Eddy KE, Vogel JP, Harrison CL, Callander E (2023). Systematic review on the cost and cost-effectiveness of mHealth interventions supporting women during pregnancy. Women Birth.

[126] Cabrera NJ, Volling BL, Barr R (2018). Fathers are parents, too! Widening the lens on parenting for children's development. Child Dev Perspect.

[127] Cooray N, Sun SL, Ho C, Adams S, Keay L, Nassar N, Brown J (2021). Toward a behavior theory-informed and user-centered mobile app for parents to prevent infant falls: development and usability study. JMIR Pediatr Parent.

[128] Pilkington PD, Whelan TA, Milne LC (2020). A review of partner‐inclusive interventions for preventing postnatal depression and anxiety. Clin Psychol.

[129] Franco LM, Pottick KJ, Huang C-C (2010). Early parenthood in a community context: neighborhood conditions, race-ethnicity, and parenting stress. J Community Psychol.

[130] Gonzalez C, Early J, Gordon-Dseagu V, Mata T, Nieto C (2021). Promoting culturally tailored mHealth: a scoping review of mobile health interventions in Latinx communities. J Immigr Minor Health.

