# Supplementary material for: Digital Parenting Interventions for Fathers of Infants From Conception to the Age of 12 Months: Systematic Review of Mixed Methods Studies
Source: J Med Internet Res. 2023 Jul 26;25:e43219. doi: 10.2196/43219 (PMC10413237; doi:10.2196/43219)
Supplement: Multimedia Appendix 1 [file jmir_v25i1e43219_app1.docx]

Appendix 1 - Original search strategies

Table S1. Medline search

| Database: Medline  Date: 2020/05/13 | Search words | Number of records |
| --- | --- | --- |
| #1 | exp Telecommunications/ | 90600 |
| #2 | exp Computer Systems/ | 176534 |
| #3 | exp Computers/ | 77718 |
| #4 | exp Cell Phone/ | 10500 |
| #5 | eHealth.mp. | 3707 |
| #6 | e-Health.tw,kf. | 2741 |
| #7 | online.tw,kf. | 112436 |
| #8 | on-line.tw,kf. | 26164 |
| #9 | internet*.tw,kf. | 52576 |
| #10 | website*. Tw,kf. | 26099 |
| #11 | computer*.tw,kf. | 301893 |
| #12 | mHealth.tw,kf. | 4457 |
| #13 | (smartphone* or “smartphone*”).tw,kf. | 11970 |
| #14 | app.tw,kf. | 25204 |
| #15 | apps.tw,kf. | 5866 |
| #16 | social media.tw,kf. | 11110 |
| #17 | web-base*.tw,kf. | 29993 |
| #18 | mobile*.tw,kf. | 98336 |
| #19 | exp Telemedicine/ | 27845 |
| #20 | application*.tw,kf. | 1184355 |
| #21 | exp Mobile applications/ | 5639 |
| #22 | cell* phone*.tw,kf. | 3443 |
| #23 | telehealth.tw,kf. | 4771 |
| #24 | exp Online Social Networking/ | 133 |
| #25 | exp Computers, Handheld/ | 7648 |
| #26 | exp Social Media/ | 7470 |
| #27 | exp Video Games/ | 5313 |
| #28 | text messag*.tw,kf. | 4087 |
| #29 | SMS.tw,kf. | 5931 |
| #30 | Computer Communication Networks/ | 13569 |
| #31 | 1 or 2 or 3 or 4 or 5 or 6 or 7 or 8 or 9 or 10 or 11 or 12 or 13 or 14 or 15 or 16 or 17 or 18 or 19 or 20 or 21 or 22 or 23 or 24 or 25 or 26 or 27 or 28 or 29 or 30 | 1893721 |
| #32 | infant*.tw,kf. | 443976 |
| #33 | baby.tw,kf. | 38005 |
| #34 | newborn*.tw,kf. | 174688 |
| #35 | babies.tw,kf. | 36650 |
| #36 | “young child*”.tw,kf. | 52111 |
| #37 | infancy.tw,kf. | 63609 |
| #38 | neonate*.tw,kf. | 90556 |
| #39 | NICU.tw,kf. | 9994 |
| #40 | exp Infant/ | 1130866 |
| #41 | 32 or 33 or 34 or 35 or 36 or 37 or 38 or 39 or 40 | 1389517 |
| #42 | exp Fathers/ | 8958 |
| #43 | father*.tw,kf. | 41466 |
| #44 | paternal*.tw,kf. | 26860 |
| #45 | dad.tw,kf. | 7500 |
| #46 | dads.tw,kf. | 883 |
| #47 | stepfather*.tw,kf. | 254 |
| #48 | stepdad*.tw,kf. | 0 |
| #49 | daddy.tw,kf. | 138 |
| #50 | 42 or 43 or 44 or 45 or 46 or 47 or 48 or 49 | 73102 |
| #51 | 31 and 41 and 50 | 453 |

Table S2. Cochrane Central Register of Controlled Trials Search

| Database: EBM Reviews – Cochrane Central Register of Controlled Trials  Date: 2020/05/15 | Search words | Number of records |
| --- | --- | --- |
| #1 | exp Telecommunications/ | 5695 |
| #2 | exp computer systems/ | 5273 |
| #3 | exp computers/ | 1298 |
| #4 | exp cellular phone/ | 773 |
| #5 | eHealth.mp. | 520 |
| #6 | e-Health.mp. | 437 |
| #7 | online.mp. | 13028 |
| #8 | on-line.mp. | 45049 |
| #9 | internet*.mp. | 10716 |
| #10 | website*.mp. | 4285 |
| #11 | computer*.mp. | 48998 |
| #12 | mHealth.mp. | 992 |
| #13 | (smartphone* or “smart phone*”).mp. | 3778 |
| #14 | app.mp. | 3490 |
| #15 | apps.mp. | 777 |
| #16 | “social media”.mp. | 1096 |
| #17 | web-base*.mp. | 6841 |
| #18 | mobile*.mp. | 8831 |
| #19 | exp telemedicine/ | 2304 |
| #20 | application*.mp. | 57472 |
| #21 | exp mobile applications/ | 560 |
| #22 | “cell* phone*”.mp. | 1289 |
| #23 | telehealth.mp. | 1419 |
| #24 | exp computers/handheld/ | 265 |
| #25 | exp social media/ | 134 |
| #26 | exp video games/ | 651 |
| #27 | “text messag*”.mp. | 3582 |
| #28 | SMS.mp. | 1956 |
| #29 | exp computer communication networks/ | 3903 |
| #30 | 1 or 2 or 3 or 4 or 5 or 6 or 7 or 8 or 9 or 10 or 11 or 12 or 13 or 14 or 15 or 16 or 17 or 18 or 19 or 20 or 21 or 22 or 23 or 24 or 25 or 26 or 27 or 28 or 29 | 177722 |
| #31 | infant*.mp. | 61088 |
| #32 | baby.mp. | 4146 |
| #33 | newborn*.mp. | 27316 |
| #34 | babies.mp. | 4295 |
| #35 | “young child*”.mp. | 5664 |
| #36 | infancy.mp. | 2511 |
| #37 | neonate*.mp. | 8374 |
| #38 | NICU.mp. | 2221 |
| #39 | exp infant/ | 30920 |
| #40 | 31 or 32 or 33 or 34 or 35 or 36 or 37 or 38 or 39 | 74958 |
| #41 | exp Fathers/ | 171 |
| #42 | father*.mp. | 1482 |
| #43 | paternal*.mp. | 511 |
| #44 | dad.mp. | 229 |
| #45 | dads.mp. | 56 |
| #46 | stepfather*.mp. | 4 |
| #47 | stepdad*.mp. | 0 |
| #48 | daddy.mp. | 6 |
| #49 | 41 or 42 or 43 or 44 or 45 or 46 or 47 or 48 | 2033 |
| #50 | 30 and 40 and 49 | 85 |

Table S3. Embase search

| Database: Embase  Date: 2020/05/14 | Search words | Number of records |
| --- | --- | --- |
| #1 | exp Telecommunications/ | 68877 |
| #2 | exp computer system/ | 25277 |
| #3 | exp computer/ | 132607 |
| #4 | exp mobile phone/ | 27817 |
| #5 | eHealth.mp. | 3856 |
| #6 | e-Health.mp. | 3794 |
| #7 | online.mp. | 179870 |
| #8 | on-line.mp. | 35528 |
| #9 | internet*.mp. | 132643 |
| #10 | website*.mp. | 39672 |
| #11 | computer*.mp. | 1558709 |
| #12 | mHealth.mp. | 4012 |
| #13 | (smartphone* or “smart phone*”).mp. | 18631 |
| #14 | app.mp. | 35786 |
| #15 | apps.mp. | 7133 |
| #16 | “social media”.mp. | 22038 |
| #17 | web-base*.mp. | 42625 |
| #18 | mobile*.mp. | 143915 |
| #19 | exp telemedicine/ | 38574 |
| #20 | application*.mp. | 1389152 |
| #21 | exp mobile application/ | 11241 |
| #22 | “cell* phone*”.mp. | 5278 |
| #23 | telehealth.mp. | 9646 |
| #24 | exp online social network/ | 213 |
| #25 | exp social media/ | 19342 |
| #26 | exp video game/ | 3389 |
| #27 | “text messag*”.mp. | 7237 |
| #28 | SMS.mp. | 8505 |
| #29 | exp computer network/ | 14737 |
| #30 | 1 or 2 or 3 or 4 or 5 or 6 or 7 or 8 or 9 or 10 or 11 or 12 or 13 or 14 or 15 or 16 or 17 or 18 or 19 or 20 or 21 or 22 or 23 or 24 or 25 or 26 or 27 or 28 or 29 | 3318356 |
| #31 | infant*.mp. | 857374 |
| #32 | baby.mp. | 64623 |
| #33 | newborn*.mp. | 640884 |
| #34 | babies.mp. | 51167 |
| #35 | “young child*”.mp. | 63732 |
| #36 | infancy.mp. | 68357 |
| #37 | neonate*.mp. | 123329 |
| #38 | NICU.mp. | 17836 |
| #39 | exp infant/ | 973880 |
| #40 | 31 or 32 or 33 or 34 or 35 or 36 or 37 or 38 or 39 | 1347445 |
| #41 | exp father/ | 27676 |
| #42 | father*.mp. | 58393 |
| #43 | paternal*.mp. | 37925 |
| #44 | dad.mp. | 10392 |
| #45 | dads.mp. | 1251 |
| #46 | stepfather*.mp. | 283 |
| #47 | stepdad*.mp. | 0 |
| #48 | daddy.mp. | 172 |
| #49 | 41 or 42 or 43 or 44 or 45 or 46 or 47 or 48 | 98816 |
| #50 | 30 and 40 and 49 | 804 |

Table S4. CINAHL search

| Database: Ebsco  Date: 2020/05/14 | Search words | Number of records |
| --- | --- | --- |
| #1 | (MH “Telecommunications+”) OR (MH “Telehealth+”) | 140899 |
| #2 | (MH “Computer Systems+” | 503061 |
| #3 | (MH “Computer Communication Networks+”) OR (MH “Email”) OR (MH “Online Services”) | 166197 |
| #4 | (MH “Video Games+”) OR (MH “Virtual Reality+”) OR (MH “Artificial Intelligence+”) OR (MH “Computer Simulation+”) | 44386 |
| #5 | TI ( eHealth OR e-Health OR online OR on-line OR internet* OR website* OR computer* OR mHealth OR smartphone* OR "smart phone*" OR app OR apps OR "social media" OR web-base* OR mobile* OR application OR "cell* phone*" OR telehealth OR "text messag* OR SMS) ) OR AB ( eHealth OR e-Health OR online OR on-line OR internet* OR website* OR computer* OR mHealth OR smartphone* OR "smart phone*" OR app OR apps OR "social media" OR web-base* OR mobile* OR application OR "cell* phone*" OR telehealth OR "text messag* OR SMS) ) | 438998 |
| #6 | 1 or 2 or 3 or 4 or 5 | 1046486 |
| #7 | (MH "Infant+") OR (MH "Infant Behavior") |  |
| #8 | TI infant* OR AB infant* |  |
| #9 | TI infancy OR AB infancy |  |
| #10 | TI (newborn* OR neonate*) OR AB (newborn* OR neonate*) | 50103 |
| #11 | TI baby OR AB baby |  |
| #12 | TI babies OR AB babies |  |
| #13 | TI ("young child*") OR AB ("young child*") | 21770 |
| #14 | TI NICU OR AB NICU | 7394 |
| #15 | 7 or 8 or 9 or 10 or 11 or 12 or 13 or 14 | 346345 |
| #16 | (MH “Fathers+”) | 7471 |
| #17 | (MH "Paternal Behavior") OR (MH "Paternal Attitudes") OR (MH "Paternal Role") OR (MH "Paternal Age") | 2505 |
| #18 | TI ( father* OR paternal* OR dad OR dads OR stepfather* OR stepdad* OR daddy ) AND AB ( father* OR paternal* OR dad OR dads OR stepfather* OR stepdad* OR daddy ) | 4640 |
| #19 | 16 or 17 or 18 | 10709 |
| #20 | 6 and 15 NS 19 | 576 |

Note:

The search strategies above were re-run in May/June 2022 for updates results prior to manuscript submission. The updated search resulted:

-221 new articles from Embase

-48 new articles from PsycInfo

-32 new articles from Medline

-66 new articles from CINAHL

-28 new articles from Cochrane
