# Supplementary material for: Digital Parenting Interventions for Fathers of Infants From Conception to the Age of 12 Months: Systematic Review of Mixed Methods Studies
Source: J Med Internet Res. 2023 Jul 26;25:e43219. doi: 10.2196/43219 (PMC10413237; doi:10.2196/43219)
Supplement: Multimedia Appendix 2 [file jmir_v25i1e43219_app2.docx]

Appendix 2 **-** Intervention descriptions organized by date of publication.

| Study | Intervention description | Exclusively eHealth^a^ | Personalized | Website | Mobile app | SMS text messaging | Social media | Web-based video | Email | Videoconference |
| --- | --- | --- | --- | --- | --- | --- | --- | --- | --- | --- |
| Hudson et al [72] | The New Fathers Network was an online intervention targeting first-time fathers that consisted of 3 different sections. The first section provided a library of information about infant growth and development, infant care, infant health, and concerns of new fathers. The second section included asynchronous discussion forms to facilitate an exchange of information, advice, and support among new fathers and advanced practice nurses. The final section included email access to advanced practice nurses. For the purposes of the study, participants in the intervention group were instructed to use the website for at least 20 minutes per week for 4 weeks. | ✓ |  | ✓ |  |  |  |  | ✓ |  |
| Fletcher et al [76] | An information package on infant health and development and parenting was mailed to expecting fathers. In total, 3 text-only emails with useful websites and information were also sent to fathers based on their topics of interest. | ✓ | ✓ | ✓ |  |  |  |  | ✓ |  |
| Salonen et al [73,74] | Online intervention based on self-efficacy theory [106] with access to an information database and an online peer discussion forum with a Q&A^b^ service provided by specially trained nurses and midwives. The online database contained information about everyday parent-child interactions, how to be sensitive to infants’ cues and needs, and how to respond to them in a growth-fostering way. Parents could use these services based on their individual needs in addition to standard care (guidance given at the hospital after childbirth and through to 1 year post partum). Furthermore, in addition to the discussion forum, parents could individually contact a trained registered nurse or midwife for 2 weeks post partum and ask anonymous questions online. | ✓ |  | ✓ |  |  |  |  |  |  |
| Benzies et al [99] | The video self-modeling with feedback intervention was based on bioecological theory [107] and self-modeling theory [108]. The video self-modeling with feedback was delivered during in-person home visits with a booster dose being provided via fathers being encouraged to watch the video interaction through a web-based platform. The intervention consisted of video recording a structured play interaction between the father and their infant aged 4 months. Following this, the father and home visitor reviewed the video, with the visitor indicating behaviors that fostered development, reinforcing the father’s strengths, and making suggestions for improvement. The structured play and feedback took 15-20 minutes, after which the father was provided with a handout addressing infant cues. For the 2-visit group, play and feedback were repeated during a home visit when infants were aged 6 months, and handouts about the teaching loop and tips for language stimulation were provided. In the 4-visit intervention group, the additional 2 visits occurred when infants were aged 5 and 7 months, and handouts about promoting language development were provided. |  | ✓ |  |  |  |  | ✓ |  |  |
| Rhoads et al [94] | This intervention consisted of online, password-protected access to web cameras that allowed parents to view their hospitalized neonates in real time at any time of day or night. | ✓ |  | ✓ |  |  |  |  |  |  |
| Garfield et al [104] | The NICU-2-Home mobile app was designed using the self-efficacy theory by Bandura [109] to support parents of VLBW^c^ infants. It consisted of the following four features: (1) Passport-2-Home, a self-guided discharge checklist; (2) an Education Center with multimedia educational information on NICU^d^ infant care; (3) Baby Connect, a commercially available app for tracking activities of daily living; and (4) a mood tracker (synchronized updates of parents’ current mood). The intervention also included a Resource Center for parents to access useful health website links, information about the app, and a list of common questions about infants in the NICU to spur conversations with the health care team. Parents could also access notes to keep track of questions and concerns about their infant. | ✓ |  | ✓ | ✓ |  |  |  |  |  |
| White et al [39,90,91] | The Milk Man mobile app was designed and developed using social cognitive theory [110] to educate new and expecting fathers on breastfeeding, infant feeding, early parenting, and being a supportive partner. Components of the app included push notifications, a forum for fathers to have conversations with one another, an information library, and gamification. Push notifications alerted users to new discussion topics, which were posted twice a week on the app. These posts allowed users to connect with each other through a series of topics initiated by the app administration. Users could interact by adding comments, upvoting, or responding to polls. An information library was also included and provided external website links. For gamification, the app used leaderboards, badges, and points to encourage engagement with both the discussion topics and information library. Fathers were grouped together based on the stage of their partner’s pregnancy. | ✓ |  | ✓ | ✓ |  |  |  |  |  |
| Fletcher et al [77-79, 82-84,111] and Lanning et al [85]^e^ | SMS4Dads is an automated program that sends SMS text messages to fathers across the prenatal and postpartum periods. Messages consisted of a maximum of 160 characters and provided information on physical health, mental health, supporting mothers through pregnancy, and building a relationship with their child. Along with these informative messages, website links to useful resources in parenting and self-care were also included. The SMS text message content and schedule were tailored to gestational age so that fathers received messages that were most relevant to them at that moment. | ✓ | ✓^e^ | ✓^e^ |  | ✓ |  |  |  |  |
| Abbass-Dick et al [89,92]^f^ | The eHealth breastfeeding coparenting resource provided information about breastfeeding and coparenting to pregnant or new mothers and their partners. The content was divided into 7 main topics that were delivered through a website. Topics covered (1) the benefits of breastfeeding, (2) how to breastfeed, and (3) expectations for breastfeeding during the early days. Another topic was oriented toward fathers, partners, and coparents and provided information on (4) supporting the mother, working as a team, involvement with breastfed children, effective communication, and problem-solving. The other topics touched on common concerns regarding (5) breastfeeding, (6) where to get help, and (7) various areas of everyday living. Information on all 7 topics was presented to users through a combination of text, quizzes, games, videos, and links to additional online resources. Users were able to select topics at their convenience. | ✓ |  | ✓ |  |  |  |  |  |  |
| Pilkington et al [69] | Partners to Parents was an online intervention that aimed to prevent perinatal depression and anxiety by facilitating partner support. This was done by providing information on the following topics via a website: potential changes during parenthood, effective communication strategies, intimacy, instrumental support, social support, establishing boundaries, self-care, depression and anxiety symptoms, and accessing professional support. For the purpose of the study, participants were asked to use the website for a single 30-minute session. | ✓ |  | ✓ |  |  |  |  |  |  |
| Da Costa et al [46] | The needs assessment in this paper will help guide the development of Healthydads.ca. Healthydads.ca will be a website with the goal of enhancing mental health and healthy behaviors in expecting fathers that can be easily accessed for resources and help on emotional wellness and pregnancy and parenting information. | ✓ |  | ✓ |  |  |  |  |  |  |
| Yu et al [96] | The intervention consisted of 3 educational home visits and supportive SMS text messages. During the home visits, participants received in-person counseling from trained health care workers on the harms of SHS^g^ to infants and a manual on establishing a smoke-free home. The smoke-free home manual provided a five-step plan: (1) deciding to create a smoke-free home, (2) talking to family members, (3) setting a date for going smoke-free, (4) actually creating a smoke-free home, and (5) keeping the home smoke-free. Mothers and their husbands received SMS text messages on the harms of SHS, health effects of smoking, and health effects of quitting. The husbands received additional SMS text messages about cessation to encourage them to quit smoking. |  |  |  |  | ✓ |  |  |  |  |
| Mackert et al [80] | The intervention consisted of slideshows presented on a website that detailed week-by-week fetal development. One slideshow used common fruits and vegetables as visuals to illustrate fetal growth (similar to widely downloaded pregnancy apps and web pages), and another slideshow used common sports objects to illustrate week-by-week growth. The slideshows were delivered via a web application where participants could access a module’s content by clicking on the illustrative icon, title, or description on the home page. | ✓ |  | ✓ |  |  |  |  |  |  |
| Lavin Venegas et al [81] | The BeSweet2Babies video (https://www.youtube.com/watch?v=L43y0H6XEH4) was a 5-minute online video that demonstrated effective infant pain management strategies (ie, breastfeeding, administration of sugar water, and kangaroo care) that parents could use when their infant experienced painful procedures such as blood tests and injections. | ✓ |  |  |  |  |  | ✓ |  |  |
| Feinberg et al [75] | In the online version of the Family Foundations intervention, couples received 5 prenatal and 3 postnatal online modules focused on helping couples consider and adjust expectations; adopt a realistic vision and prepare for the strains of parenthood; and develop skills related to supportive, cohesive parenting communication and problem-solving. In between the modules, written and communication exercises were presented for the couples to complete. After starting the program, if the couple stopped engaging with it for >10 days, email reminders were sent encouraging them to continue. The development of the intervention was partly based on the innovative group discussion approach to supporting couples in the transition to parenthood developed by Philip Cowan and Carolyn Cowan. | ✓ |  | ✓ |  |  |  |  | ✓ |  |
| Bonifácio et al [95] | The PRENACEL^h^ program is an SMS text messaging intervention that provided information about prenatal care to partners of pregnant women and encouraged partner involvement during pregnancy. The intervention included a total of 62 SMS text messages delivered during gestation weeks 5-42 and the immediate postpartum period. Partners received 1 to 2 SMS text messages each week, with the content related to the pregnant woman’s gestational age. The messages were adapted from the MAMA^i^. | ✓ | ✓ |  |  | ✓ |  |  |  |  |
| Missler et al [97] | This psychoeducational intervention aimed to prevent postpartum parenting stress and enhance parental well-being and caregiving quality. Delivery of the intervention consisted of an information booklet, an online video, a home visit, and a telephone call. The booklet contained four chapters outlining the following topics: (1) infants’ needs and signals of distress, (2) patterns of crying and soothing techniques, (3) hunger signals and feeding arrangements, and (4) sleeping patterns and sleeping arrangements. The online video illustrated these topics and engaged participants in thinking about how they could implement the information in their own lives, which took 15-20 minutes. The home visit was then used to discuss the material further and respond to any questions from parents. A phone call check-in at 4 weeks post partum was used to ask parents how they were doing and discuss any challenges. |  |  |  |  |  |  | ✓ |  |  |
| Firouzan et al [70] | There were 2 intervention groups. In one intervention group, fathers received in-person training via speech, image display, sharing of experiences, and Q&A over two 2-hour sessions. They then received 3-4 SMS text messages weekly up to 3 months about pregnancy, the partner’s role in perinatal care, relationship with the fetus and the mother, and other preparatory information. Training content included information about changes in women’s bodies during pregnancy, common complaints during pregnancy, and the role of men in women’s adaption. It also included information about men’s participatory role in perinatal care, including the father’s relationship with the fetus and the mother; preparation for childbirth and fatherhood; how to deal with danger signs during pregnancy; the role of men in the delivery process; and social support during pregnancy, childbirth, and the postpartum period. It also addressed barriers to men’s participation during pregnancy, childbirth, and the postpartum period and remedies. The other intervention group received the same information via CD. |  |  |  |  | ✓ |  |  |  |  |
| Marcell et al [71] | This protocol described the Text4FATHER SMS text messaging program, which is informed by the Integrated Behavior Model [112] and aims to provide fathers with guidance on infant, partner, and personal well-being support. This will be done through the delivery of twice-weekly texts for 7 months that will be tailored to the mother’s gestational age, infant age after birth, and father’s resident status. Of the total 71 texts, 24 will include weblinks that share additional information, infographics, and videos. | ✓ | ✓ |  |  | ✓ |  |  |  |  |
| Manav et al [100] | Couples were part of a WhatsApp parenting group where they received education modules in the form of written SMS text messages or voice recordings. These education modules provided information regarding issues such as the baby’s biopsychosocial care (according to the baby’s developmental level) and assessment and parent-infant communication. Each voice recording message was approximately 5 to 10 minutes long. Couples could also ask questions and engage in an online consultancy with a specialist psychiatric nurse, a specialist gynecology nurse, and a specialist pediatric nurse at any time during the day in their WhatsApp group. In these groups, they also shared their experiences, problems, and solution suggestions about the postpartum period and baby care. | ✓ | ✓ |  | ✓ |  |  |  |  |  |
| Doaltabadi and Amiri-Farahani [101] | This intervention was delivered in a mobile app-based format and provided fathers with information on (1) pregnancy diet, (2) mental health during pregnancy, (3) planning for delivery and selecting the type of delivery, and (4) neonatal care. Educational content was uploaded to the social media mobile app (Telegram) and delivered to participants prenatally at 4 time points (24-28 weeks, 28-30 weeks, 32-33 weeks, and 37 weeks). | ✓ |  |  | ✓ |  | ✓ |  |  |  |
| Kavanagh et al [88] | Participants were randomized to either the Baby Steps Wellbeing (treatment) or the Baby Care web program (active control group) interventions. Both arms received four self-paced online modules with information on (1) preparing for birth, (2) infant feeding, (3) infant sleep, and (4) infant soothing, as well as a list of telephone support numbers or digital services. The Baby Steps Wellbeing group also received 5 additional online modules on self-care, their romantic relationship, baby interaction, role adjustment, and a module created especially for fathers, as well as a space for goal setting, a web-based scrapbook option, and quiz questions about baby care. Both groups received automated SMS text messages 2, 4, 7, and 10 weeks after condition allocation reminding parents to log into the program. The SMS text messages to parents in the Baby Steps Wellbeing condition also included recommendations to review their goals. | ✓ |  | ✓ |  | ✓ |  |  |  |  |
| Scott et al [93] | The digital interventions included the Milk Man smartphone app–only group (see the aforementioned description [39]) as well as a combination group that included access to the Milk Man app in addition to a face-to-face father-focused antenatal breastfeeding class, which was facilitated by a male peer. Both groups had access to the app from approximately week 32 of gestation to 6 months post partum. |  |  | ✓ | ✓ |  |  |  |  |  |
| Hägi-Pedersen et al [87] | Parents in the intervention group were enrolled in an early in-home care program that included video consultations with a neonatal nurse. Planned video consultations with the nurse occurred 2-3 times a week. During these consultations, parents received information on early in-home care and learned skills related to in-home care. | ✓ |  |  |  |  |  |  |  | ✓ |
| Shorey et al [86] | The SPA^j^ was an online intervention delivered through a mobile app. It was developed based on social cognitive theory [110] and attachment theory [114]. The main features of the intervention included knowledge-based content, informational videos and audio clips, an online discussion forum, group and private chats with peer volunteers, expert advice from a maternity unit nurse or midwife through an online forum, and individualized push notifications. Information was provided on the topics of pregnancy, childbirth, baby care, maternal care, family, and parenthood. The intervention included 42 knowledge-based articles and 27 inspirational and demonstration videos. Push notifications were sent on a weekly basis during pregnancy, on a daily basis for 1 month post partum, and biweekly following that until 6 months after childbirth. | ✓ |  |  | ✓ |  |  | ✓ |  |  |
| Whooten et al [103] | The First Heroes program was developed using the Chronic Care Model and is a blended intervention delivered through a series of 3 virtual visits and engagement with informational content. The virtual visits were conducted by a trained health educator/behavioral health coach, with the first visit occurring at 30-34 weeks of gestation, the second occurring at 3-4 weeks post partum, and the third occurring at 3-4 months post partum. Additional informational content was delivered through print and email materials, videos, and SMS text messages. The SMS text messages were sent at a frequency of 1-3 messages per week from 22 weeks of gestation to a year post partum. The information provided discussed maternal and paternal health, social-emotional wellness, responsive parenting practices, and social determinants of health and access to community resources. |  |  |  |  | ✓ |  | ✓ | ✓ | ✓ |
| Giuseppe et al [105] | In the T-FCC^k^ group, parents were not able to access the NICU but were allowed to see their newborns via smartphone video calls. Video calls occurred while newborns were feeding or sleeping or during procedures. During this time, parents were updated on the process of care and provided with support from a psychologist on the call. Parents were also able to receive telephone updates on the clinical status of their newborns. | ✓ | ✓ |  |  |  |  |  |  | ✓ |
| Zhang et al [98] | Couples with an infant with CHD^l^ received health education and care guidance via WeChat. The intervention included 2 parts—the education module and the Q&A module. The education module included related knowledge on CHD, postoperative care, family care, feeding, and management of complications. Parents could view the module and learn at any time that was convenient for them. The Q&A module included a medical staff member that was available via WeChat to address parents’ problems and guide the family members to discuss and share their care experiences. | ✓ | ✓ |  | ✓ |  |  |  |  |  |
| Park and Bang [102] | Fathers received 5 intervention sessions over a 5-week period. The first and fifth sessions involved home visits (30 minutes each) where the fathers were individually educated by the researcher on how to interact with their infants. Written feedback on father-infant interactions was then provided. Over the course of 5 weeks, 5 online interactive videoconferencing sessions were conducted on the same topic. The first and fifth sessions lasted 30 minutes each, whereas the other 3 took 60 minutes each. The online lecture content focused on developing the caregiver’s sensitivity, building infant developmental knowledge, and recognizing the teaching loop during father-infant interactions. |  | ✓ |  |  |  |  |  |  | ✓ |

^a^*Exclusively* *eHealth* indicates that there was no in-person contact with a human provider. Pilot studies include feasibility and usability testing.

^b^Q&A: question and answer.

^c^VLBW: very low birth weight.

^d^NICU: neonatal intensive care unit.

^e^Fletcher et al [82-84,78,79,111] included a mood tracker as part of the intervention. Fletcher et al [84] and Lanning et al [85] were designed specifically for partners of mothers with severe mental illness (the content of the SMS text messages was tailored specifically for this purpose) and did not mention including any links to helpful websites. Lanning et al [85] and Fletcher et al [77,84] did not report the interventions being personalized based on gestational age. The timing and frequency of the messages varied across the studies.

^f^Abbass-Dick et al [92] condensed the 2 separate everyday living sections into 1 section and included sections on where to obtain help and helpful links.

^g^SHS: secondhand smoking.

^h^PRENACEL: bi-directional, mobile-phone based, short text message service.

^i^MAMA: Mobile Alliance for Maternal Action.

^j^SPA: Supportive Parenting App.

^k^T-FCC: Telematic Family-Centered Care.

^l^CHD: congenital heart disease.
