# Supplementary material for: Digital Parenting Interventions for Fathers of Infants From Conception to the Age of 12 Months: Systematic Review of Mixed Methods Studies
Source: J Med Internet Res. 2023 Jul 26;25:e43219. doi: 10.2196/43219 (PMC10413237; doi:10.2196/43219)
Supplement: Multimedia Appendix 3 [file jmir_v25i1e43219_app3.docx]

Appendix 3 – Mixed Methods Appraisal Tool (MMAT) quality assessment tables

Table S1**.** MMAT quality appraisal for qualitative study designs

|  | Fletcher, Knight et al., 2019 | Fletcher et al., 2020 | Pilkington et al., 2017 | Hägi-Pedersen et al., 2021 | White et al., 2018 | Lanning et al., 2021 |
| --- | --- | --- | --- | --- | --- | --- |
| Approach appropriate? | Y | Y | Y | Y | Y | Y |
| Data collection methods adequate? | Y | Y | Y | Y | Y | Y |
| Findings adequately derived from data? | Y | Y | Y | Y | Y | Y |
| Interpretation sufficiently substantiated by data? | Y | N | Y | Y | Y | Y |
| Coherence between qualitative data courses, collection, analysis, and interpretation? | Y | Y | Y | Y | Y | Y |

Table S2. MMAT quality appraisal for quantitative RCT study designs

|  | Bonifacio et al., 2020 | Venegas et al., 2019 | Benzies et al., 2013 | Yu et al., 2017 | Kavanagh et al., 2021 | Scott et al., 2021 | Missler et al., 2020 | Garfield et al., 2016 | Manav et al., 2021 | Firouzan et al., 2021 | Zhang et al., 2021 | Feinberg et al., 2020 |
| --- | --- | --- | --- | --- | --- | --- | --- | --- | --- | --- | --- | --- |
| Randomization appropriate? | Y | Y | Y | Y | Y | Y | Y | Y | Y | N | Y | Y |
| Groups comparable at baseline? | N | Y | Y | Y | Y | Y | Y | N | Y | Y | Y | Y |
| Complete outcome data? | Y | Y | Y | Y | Y | Y | Y | Y | N | Y | Y | N |
| Outcome assessors blinded to intervention? | N | N | Y | Y | Y | N | N | N | Y | N | N | N |
| Participants adhere to assigned intervention? | N | Y | Y | Y | Y | Y | Y | Y | N | Y | N | N |

Table S3. MMAT quality appraisal for quantitative non-randomized study designs

|  | Abbass-Dick et al., 2017 | Hudson et al., 2003 | Rhoads et al., 2015 | Salonen et al., 2011 | Doaltabadi and Amiri-Farahani, 2021 | Park & Bang, 2022 | Giuseppe et al., 2022 |
| --- | --- | --- | --- | --- | --- | --- | --- |
| Participants representative of target population? | Y | N | N | N | N | N | Y |
| Outcome and intervention measures appropriate? | Y | Y | Y | Y | Y | Y | N |
| Complete outcome data? | N | N | Y | N | Y | Y | Y |
| Confounders accounted for in the design and analysis? | N | N | N | N | N | Y | N |
| Intervention administered during the study period as intended? | Y | N | Y | N | Y | Y | Y |

Table S4. MMAT quality appraisal for quantitative Description study designs

|  | Fletcher et al., 2008 | Fletcher, Kay-Lambkin et al., 2017 | White et al., 2019 | Salonen et al., 2008 | Fletcher, Campbell et al., 2019 | DaCosta et al., 2017 |
| --- | --- | --- | --- | --- | --- | --- |
| Sampling strategy relevant to address research question? | Y | Y | Y | Y | N | Y |
| Sample representative of target population? | N | Y | Y | N | N | N |
| Measures appropriate? | Y | Y | Y | N | Y | Y |
| Risk of nonresponse bias low? | N | Y | Y | Y | N | Y |
| Statistical analysis appropriate to answer research question? | Y | Y | Y | Y | N | Y |

Table S5. MMAT quality appraisal for Mixed Methods study designs

|  | Fletcher et al., 2016 | Fletcher, May et al., 2017 | Mackert et al., 2017 | White et al., 2016 | Abbass-Dick et al., 2020 |
| --- | --- | --- | --- | --- | --- |
| Adequate rationale for using mixed methods design to answer research question? | Y | Y | N | N | N |
| Components of the study effectively integrated to answer research question? | Y | Y | Y | N | N |
| Outputs of integration of qualitative and quantitative components adequately interpreted? | Y | Y | Y | N | N |
| Divergences and inconsistencies between quantitative and qualitative results adequately addressed? | Y | Y | Y | N | N |
| Different components of the study adhere to the quality criteria of each quantitative and qualitative component involved? | N | N | N | N | N |
| Number of criteria meet for quantitative and qualitative components. | 3/10 | 4/10 | 6/10 | 8/10 | 7/10 |
